# The Structure of the Barley Husk Influences Its Resistance to Mechanical Stress

**DOI:** 10.3389/fpls.2020.614334

**Published:** 2021-01-26

**Authors:** Kathryn R. Grant, Maree Brennan, Stephen P. Hoad

**Affiliations:** ^1^School of Biological Sciences, College of Science and Engineering, Institute of Plant Sciences, University of Edinburgh, Edinburgh, United Kingdom; ^2^Department of Agriculture, Horticulture and Engineering Sciences, Scotland's Rural College, Edinburgh, United Kingdom

**Keywords:** barley, crop improvement, grain quality, grain skinning, plant physiology, plant biomechanics

## Abstract

This paper explores the links between genotype, plant development, plant structure and plant material properties. The barley husk has two organs, the lemma and the palea, which protect the grain. When the husk is exposed to mechanical stress, such as during harvesting, it can be damaged or detached. This is known as *grain skinning*, which is detrimental to grain quality and has a significant economic impact on industry. This study focused on the lemma, the husk organ which is most susceptible to grain skinning. This study tested three hypotheses: (1) genotype and plant development determine lemma structure, (2) lemma structure influences the material properties of the lemma, and (3) the material properties of the lemma determine grain skinning risk. The effect of genotype was investigated by using plant material from four malting barley varieties: two with a high risk of grain skinning, two with a low risk. Plant material was assessed at two stages of plant development (anthesis, GS 65; grain filling, GS 77). Structure was assessed using light microscopy to measure three physiological features: thickness, vasculature and cell area. Material properties were approximated using a controlled impact assay and by analyzing fragmentation behavior. Genotype had a significant effect on lemma structure and material properties from anthesis. This indicates that differences between genotypes were established during floral development. The lemma was significantly thinner in high risk genotypes, compared to low risk genotypes. Consequently, in high risk genotypes, the lemma was significantly more likely to fragment. This indicates a relationship between reduced lemma thickness and increased fragmentation. Traditionally, a thin husk has been considered beneficial for malting quality, due to an association with malt extract. However, this study finds a thin lemma is less resistant to mechanical stress. This may explain the differences in grain skinning risk in the genotypes studied.

## 1. Introduction

Barley (*Hordeum vulgare*) is the fourth largest cereal crop grown worldwide. It is economically important due to its role in animal feed, human consumption and the malting, brewing, and distilling industry (Baik and Ullrich, 2008). The malting, brewing, and distilling industry is a particularly high-value market, but requires barley grain to meet stringent quality specifications. Barley grain quality is assessed using a number of traits, such as grain size, composition or enzymatic properties, many of which are determined during plant development (Kumlehn and Stein, 2014). This study focuses on a specific grain quality trait, known to the malting sector as grain skinning, which measures the loss of the barley husk. Grain skinning has been reported for decades (Harlan and Martini, 1936).

The barley husk is formed from two floral structures unique to the grasses, the lemma and palea. Unlike other cereals, the barley husk adheres to the barley seed, known as a caryopsis, during grain development. Once the grain is mature, the adhesion between husk and caryopsis is so strong, they cannot be separated (Evers and Millar, 2002; Hoad et al., 2016). This is an intriguing example of adhesion in a biological system.

The lemma and palea are non-homologous structures. Each organ is controlled by distinct genetics and developmental pathways (Pozzi et al., 2000; Luo et al., 2005; Yuan et al., 2009). The lemma is derived from the leaf subtending the axillary meristem. The palea is derived from two fused prophylls (Kellogg, 2001). Both organs differ in their anatomy. The palea is smaller, covers the ventral crease, and has two vascular bundles. The lemma is larger, overlapping the palea slightly at the lateral portions of the grain, and has five vascular bundles. Both organs have a similar tissue organization, with four cell layers: outer epidermis, sclerenchyma, parenchyma and inner epidermis. The outer epidermal and sclerenchymal cells have thick, rigid cell walls, which strengthen the husk. The parenchymal and inner epidermal cells are large, with thin cell walls. The vascular bundles are embedded in the parenchyma. The inner epidermis of each organ adheres to the caryopsis (Gaines et al., 1985; Evers and Millar, 2002; Olkku et al., 2005; Gubatz et al., 2007; Kohl et al., 2015).

The husk protects the caryopsis from biological, environmental and mechanical stresses. It also maintains an important role in maintaining seed dormancy. Malting is a process of controlled germination and industry relies on highly consistent levels of germination (Gupta et al., 2010). Losing the husk results in increased damage to the embryo, uneven germination and poor endosperm modification. High proportions of skinned grains are therefore detrimental to malting efficiency (Meredith, 1959; Agu et al., 2002; Hoad et al., 2016; Okoro et al., 2017).

Grain skinning can be triggered by mechanical stress and is first observed when the crop is harvested. Highly abrasive combine harvester settings, such as a high drum speed and low concave setting, have been shown to increase grain skinning during harvesting. Grain skinning then escalates with further handling, as the crop progresses through the malting, brewing, and distilling pipeline (Hoad et al., 2003; Olkku et al., 2005). Husk loss is also influenced by genotype and environmental conditions, such as rainfall and temperature (Brennan et al., 2017a,b, 2019).

The mechanism underlying grain skinning is unknown. Logic suggests there are two possible hypotheses. The adhesion between the husk and the caryopsis may fail, allowing the husk to be separated from the caryopsis. This would be an example of “adhesion failure” in a biological system. Alternatively, failure could occur within the husk or caryopsis tissue, allowing the husk to be separated from the caryopsis. This would be an example of “substrate failure”—to use terminology from adhesion mechanics (Kinloch, 2012)—in a biological system.

Most research has investigated the hypothesis that adhesion failure causes grain skinning (Hoad et al., 2016; Brennan et al., 2017a,b, 2019). Adhesion between the husk and caryopsis is established during grain development, between developmental growth stage (GS) 75 and GS 85 (Gaines et al., 1985; Taketa et al., 2008; Duan et al., 2015; Hoad et al., 2016). A cascade of transcriptional changes occur around GS 75, many relating to the biosynthesis, transport and regulation of cuticular compounds. The cuticle of the pericarp, the outermost layer of the caryopsis, increases in thickness and develops a unique modification, known as the “cementing layer.” In addition to a change in cuticle structure, there appears to be complex changes in cuticle composition (Duan et al., 2015; Brennan et al., 2019). Subsequently, at GS 77, there is a marked increase in pericarp cuticle permeability (Taketa et al., 2008; Duan et al., 2015; Grant et al., in preparation). This is significant, as increased cuticle permeability has been implicated in adhesion or organ fusion in a range of plant species (Yephremov et al., 1999; Sieber et al., 2000; Smirnova et al., 2013; Yeats and Rose, 2013). A functional NUD gene, which encodes an ethylene responsive transcription factor, is necessary, although not sufficient, for the development of the cementing layer and a permeable pericarp cuticle (Taketa et al., 2008; Kakeda et al., 2011). Without these modifications to the pericarp cuticle, there can be no adhesion between the barley caryopsis and the barley husk, or the adhesion is critically impaired in some way. Thus, when the barley *nud* mutant is threshed, the husk is lost, leaving behind a naked caryopsis (Gaines et al., 1985; Taketa et al., 2008; Duan et al., 2015). There are key differences between the *nud* phenotype and the grain skinning phenotype. In malting barley varieties, the husk exhibits husk damage and partial husk loss, rather than complete husk loss. It has been hypothesized that grain skinning in malting barley varieties is caused by a less severe version of the *nud* phenotype. Consequently, previous research has investigated whether environmental conditions or certain genotypes result in a *nud*-like pericarp cuticle, leading to impaired adhesion, followed by husk loss (Hoad et al., 2016; Brennan et al., 2017a,b, 2019).

Previous research has not investigated the alternative hypothesis to adhesion failure: that failure within the husk or caryopsis tissue results in grain skinning. This is worthy of a greater research focus. Grains are subjected to significant mechanical forces during harvesting and processing. It is highly likely that the husk tissue experiences significant stress. Failure within the husk tissue is therefore a plausible cause of husk damage, husk loss and grain skinning.

This study was designed to test three hypotheses: (1) genotype and plant development determine the structure of the husk, (2) husk structure influences the material properties of the husk, and (3) the material properties of the husk influence the probability of grain skinning occuring.

This study focused specifically on the lemma, as Grant (2019) demonstrated that the lemma was eight times more likely to be affected by grain skinning than the palea. The lemma is particularly important for grain quality, because it covers and protects the embryo.

To examine the effect of genotype, four malting barley varieties were used. Two varieties, Propino and Poker, are known to have a high probability, or risk, of grain skinning in the field, whereas two other varieties, Henni and Golden Promise, are known to have a low risk of grain skinning (Brennan et al., 2017b, 2019; Grant, 2019). Plant material was collected from these varieties at two stages of development (GS 65, GS 77).

Previous work in plants have shown that several structures can influence material properties: tissue thickness, tissue density, cellular organization and vasculature (Speck and Burgert, 2011; Gibson, 2012; Faisal et al., 2013; Brulé et al., 2016; Shah et al., 2017; Geitmann and Gril, 2018). This study therefore measured these structures in the lemma, using light microscopy, to determine whether structure influenced material properties. Specifically, this study measured: lemma thickness, vascular bundle diameter and sclerenchymal cell area. This is discussed further in the Methods and Discussion.

Husk material properties were estimated from fragmentation in this study. The barley husk is small, has a complex morphology and delicate tissue. This made it very difficult to measure specific material properties, such as strength, stiffness or toughness, using material science methods. Instead, the barley lemma was exposed to mechanical stress in the form of a controlled impact assay. Impact is a common form of mechanical stress experienced by grains during harvesting and processing. Impact caused the lemma to fragment; fragment number and area were used as indicators of overall material properties. This assay was sufficient to demonstrate that there are biologically significant differences in the material properties of the lemma. These differences are likely to have real-world implications for the industrial processing of barley grains.

The relationship between genotype, structure and material properties were examined. Genotypes with a high risk of grain skinning were expected to form either a greater number of fragments or fragments with a smaller in area. It was expected that these differences in material properties were caused by underlying differences in structure.

## 2. Results

### 2.1. Husk Structure

#### 2.1.1. Thickness

Thickness was measured at three points along the longitudinal axis of the lemma: in the central, mid and lateral regions. Minimum and maximum thickness were measured in each region.

**Figure 1:**
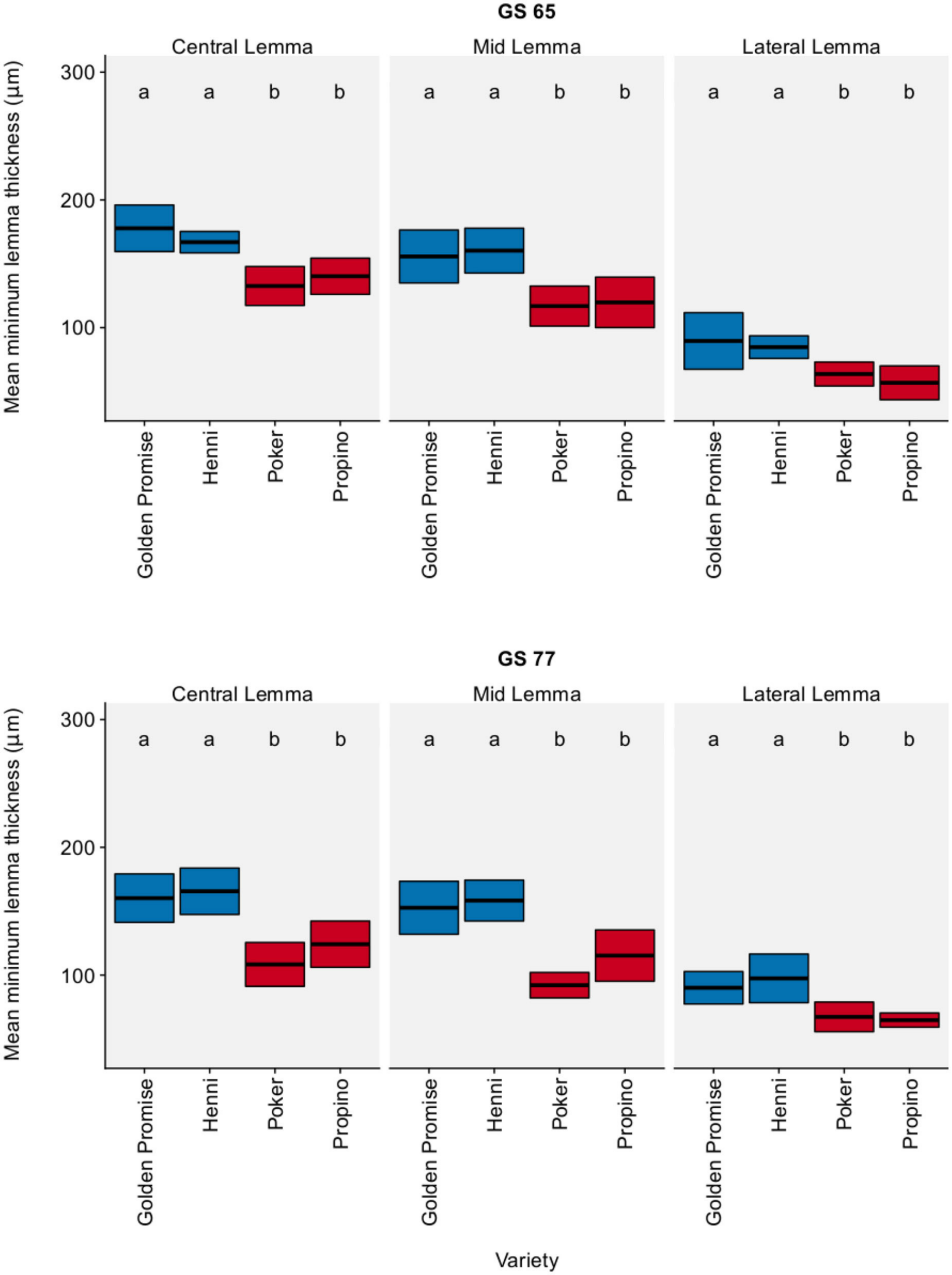
Minimum lemma thickness. Graph displays the mean thickness and 95% CI. 95% CIs calculated using $Z_{\frac{\alpha }{2}}\times$ standard error of the mean. *N* = 6 for each Golden Promise × growth stage combination; *N* = 10 for all other variety × growth stage combinations. Differences between varieties and growth stages were assessed using an ANOVA test followed by a Tukey's HSD test. This is summarized by annotating the graph with letters; varieties and growth stages which do not share a letter are significantly different from each other. High risk varieties are shown in red, low risk varieties are shown in blue.

Figure 1 shows the lemma minimum thickness by region, growth stage and variety. In all three regions of the lemma, high risk varieties were significantly thinner than low risk varieties (see also Additional Table 3, which gives a 95% CI for the pairwise differences between varieties). Overall, high risk varieties were thinner than the low risk varieties by a factor of 0.61 (95% CI = 0.51, 0.72). Variety differences were clear at both GS 65 and GS 77. In general, growth stage had no significant effect on the minimum thickness of the lemma, except for a single measurement, the minimum thickness of the central lemma (see Additional Table 4). This implies that variety differences in lemma thickness arose during floral development, not during grain development.

**Figure 2:**
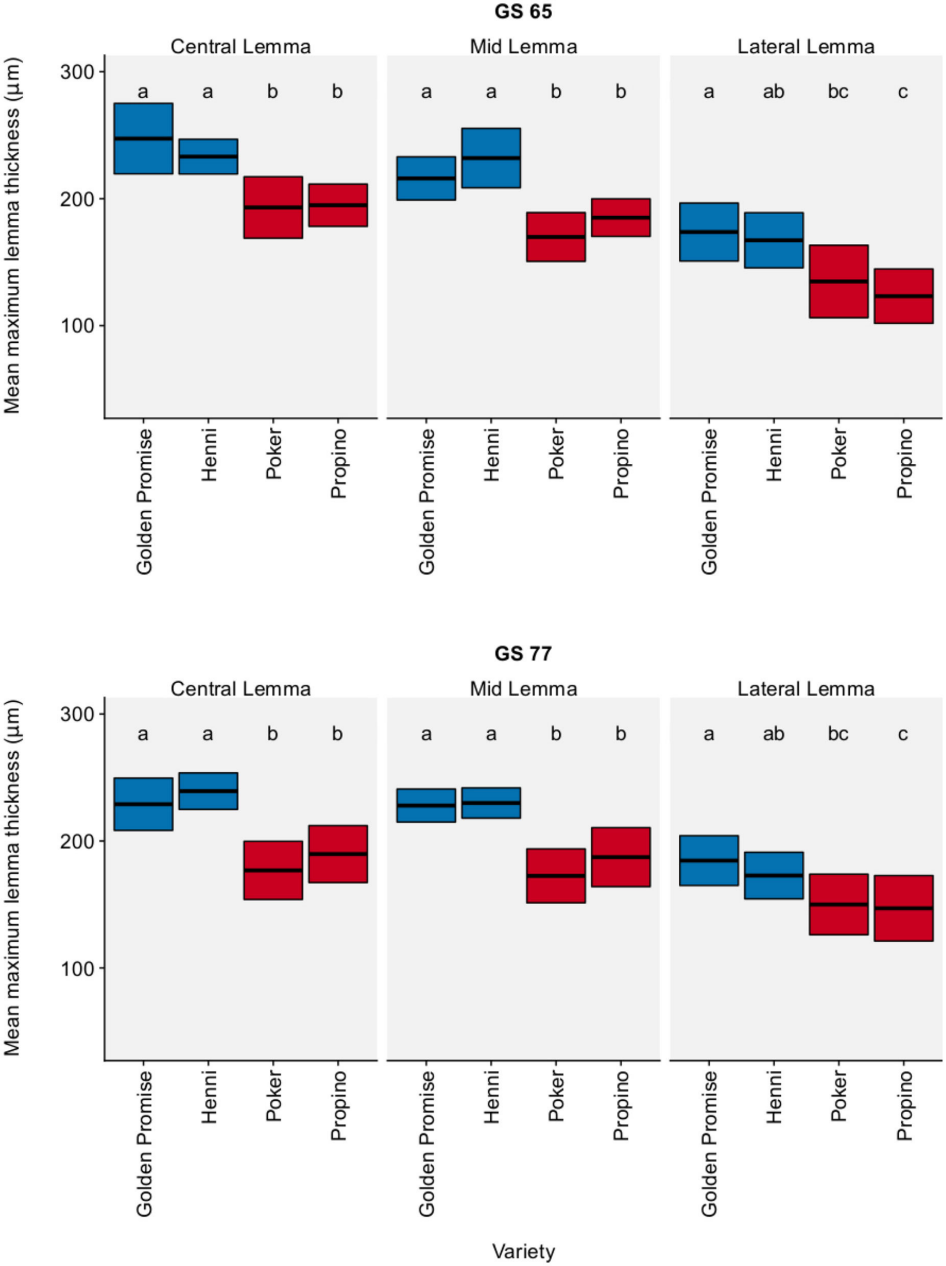
Maximum lemma thickness. Graph displays the mean thickness and 95% CI. 95% CIs calculated using $Z_{\frac{\alpha }{2}}\times$ standard error of the mean. *N* = 6 for each Golden Promise × growth stage combination; *N* = 10 for all other variety × growth stage combinations Differences between varieties and growth stages were assessed using an ANOVA test followed by a Tukey's HSD test. This is summarized by annotating the graph with letters; varieties and growth stages which do not share a letter are significantly different from each other. High risk varieties are shown in red, low risk varieties are shown in blue.

The results for maximum lemma thickness were very similar. Figure 2 shows the lemma maximum thickness by region, growth stage and variety. In all three regions of the lemma, high risk varieties were significantly thinner than low risk skinning varieties, except for one single comparison: the difference in the maximum thickness of the lateral region of the lemma was not significant between Poker and Henni (see also Additional Table 3). Overall, high risk varieties were thinner than the low risk varieties by a factor of 0.74 (95% CI = 0.67, 0.80). Variety differences were clear at both GS 65 and GS 77. There was no significant change in lemma thickness between GS 65 and GS 77, implying that differences in lemma thickness arose during floral development (see Supplementary Table 4).

In conclusion, high risk varieties had thinner lemmas than low risk varieties.

#### 2.1.2. Vascular Bundles

**Figure 3:**
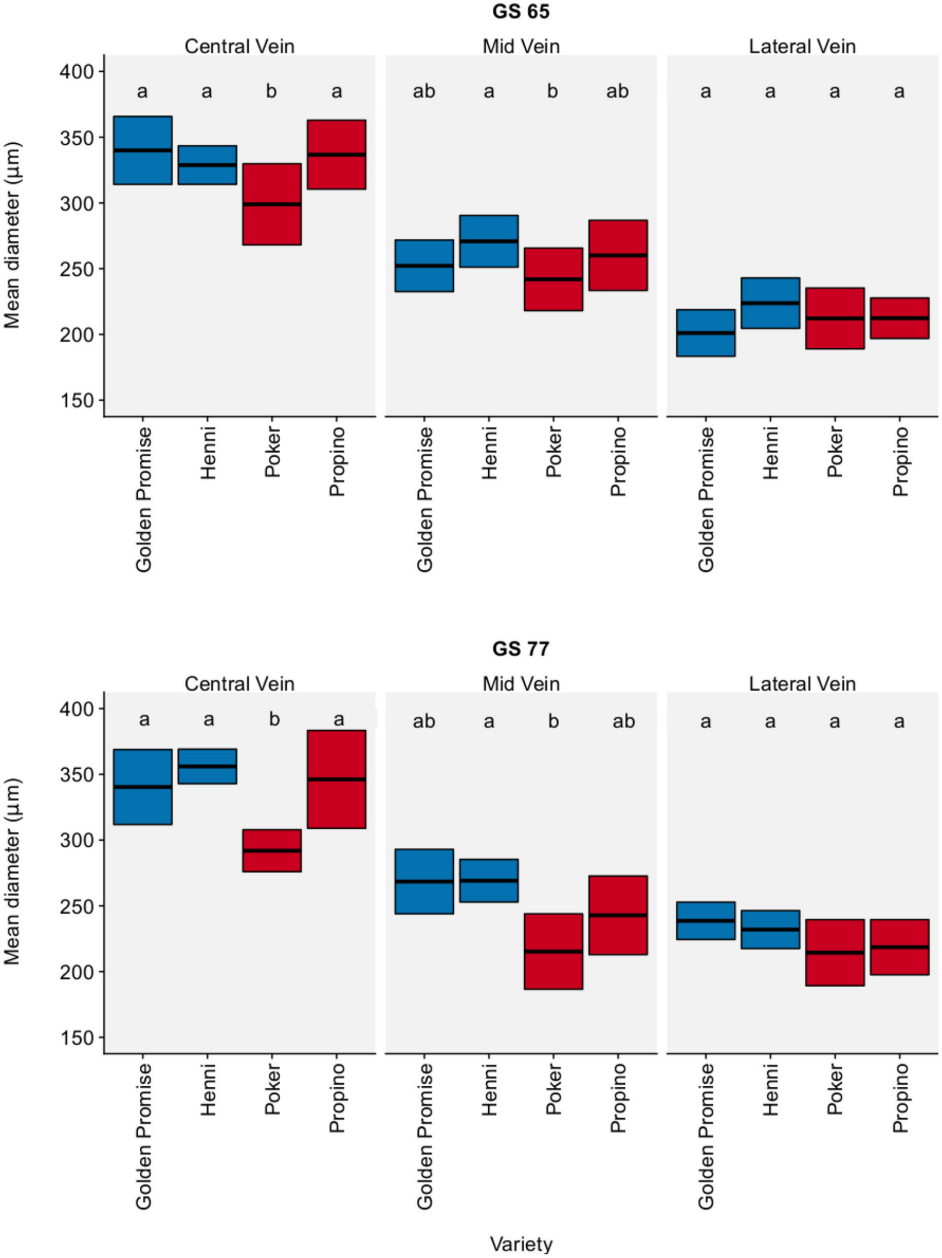
Vascular bundle diameter. Measured in the lemma. Graph displays the mean diameter and 95% CI. 95% CIs calculated using $Z_{\frac{\alpha }{2}}\times$ standard error of the mean. *N* = 6 for each Golden Promise × growth stage combination; *N* = 10 for all other variety × growth stage combinations. Differences between varieties and growth stages were assessed using an ANOVA test followed by a Tukey's HSD test. This is summarized by annotating the graph with letters; varieties and growth stages which do not share a letter are significantly different from each other. High risk varieties are shown in red, low risk varieties are shown in blue.

Figure 3 shows vascular bundle diameter in the lemma by region, growth stage and variety. Vascular bundle diameter was greatest in the center of the lemma and narrowest at the lateral edge of the lemma. Although there were significant differences between varieties, there was no clear relationship between vascular bundle diameter and fragmentation. There was also no significant change in vascular bundle diameter between GS 65 and GS 77. The pairwise differences, with a 95% CI, between varieties and growth stages are shown in Supplementary Table 6.

#### 2.1.3. Cellular Organization

**Figure 4:**
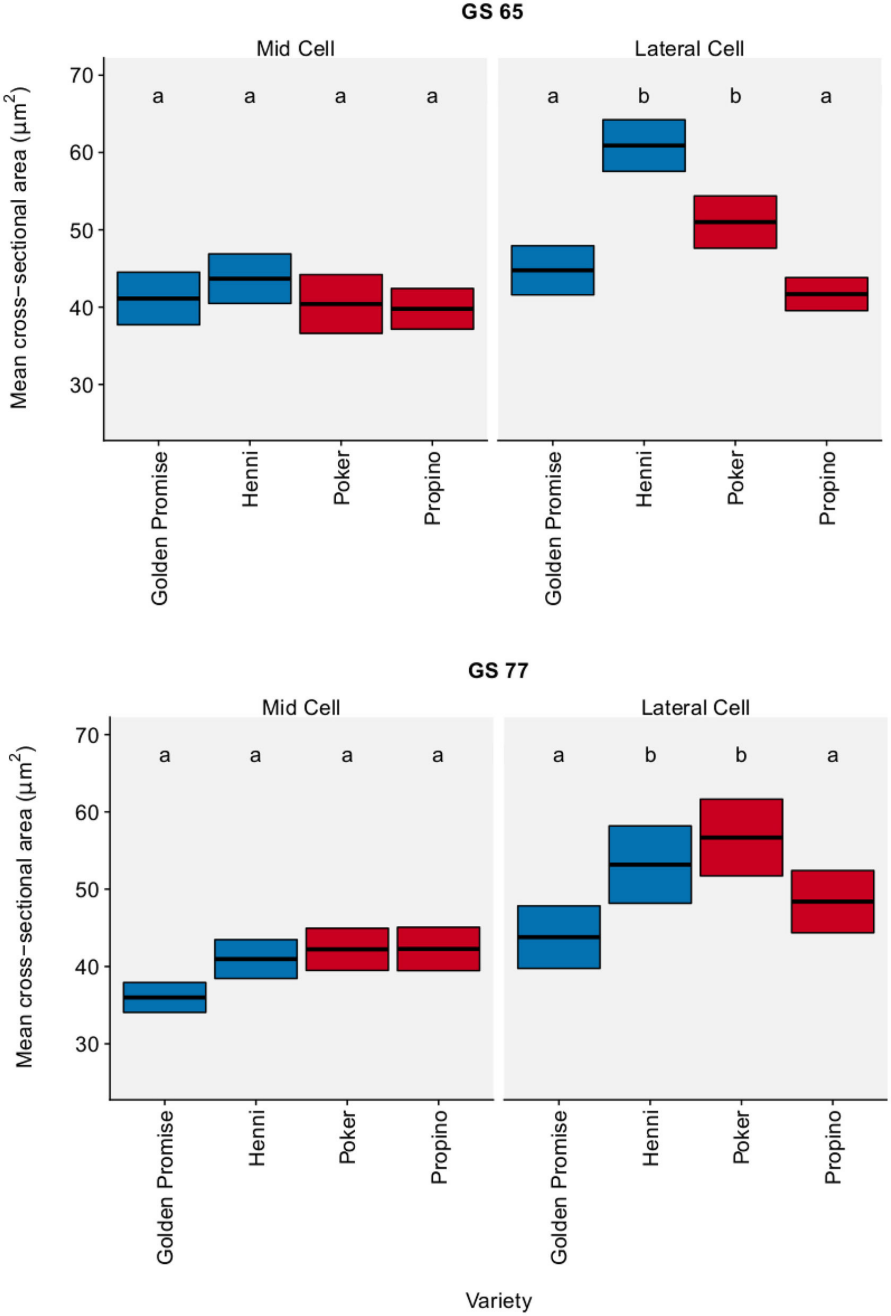
Cell cross-sectional area. Sclerenchymal cells in the lemma measured. Graph displays the mean area and 95% CI. *N* = 25 for Golden Promise; *N* = 50 for all other varieties. Differences between varieties and growth stages were assessed using an ANOVA test followed by a Tukey's HSD test. This is summarized by annotating the graph with letters; varieties and growth stages which do not share a letter are significantly different from each other. High risk varieties are shown in red, low risk varieties are shown in blue.

The cross-sectional area of sclerenchymal cells was measured in the mid region and lateral region of the lemma. Figure 4 shows cell area by region, growth stage and variety. There was no significant change in cell area between GS 65 and GS 77. Although there were significant differences between varieties, there was no clear relationship between cell area and fragmentation. The pairwise differences, with a 95% CI, between varieties and growth stages are shown in Supplementary Table 8.

### 2.2. Husk Material Properties

#### 2.2.1. Fragment Number

**Figure 5:**
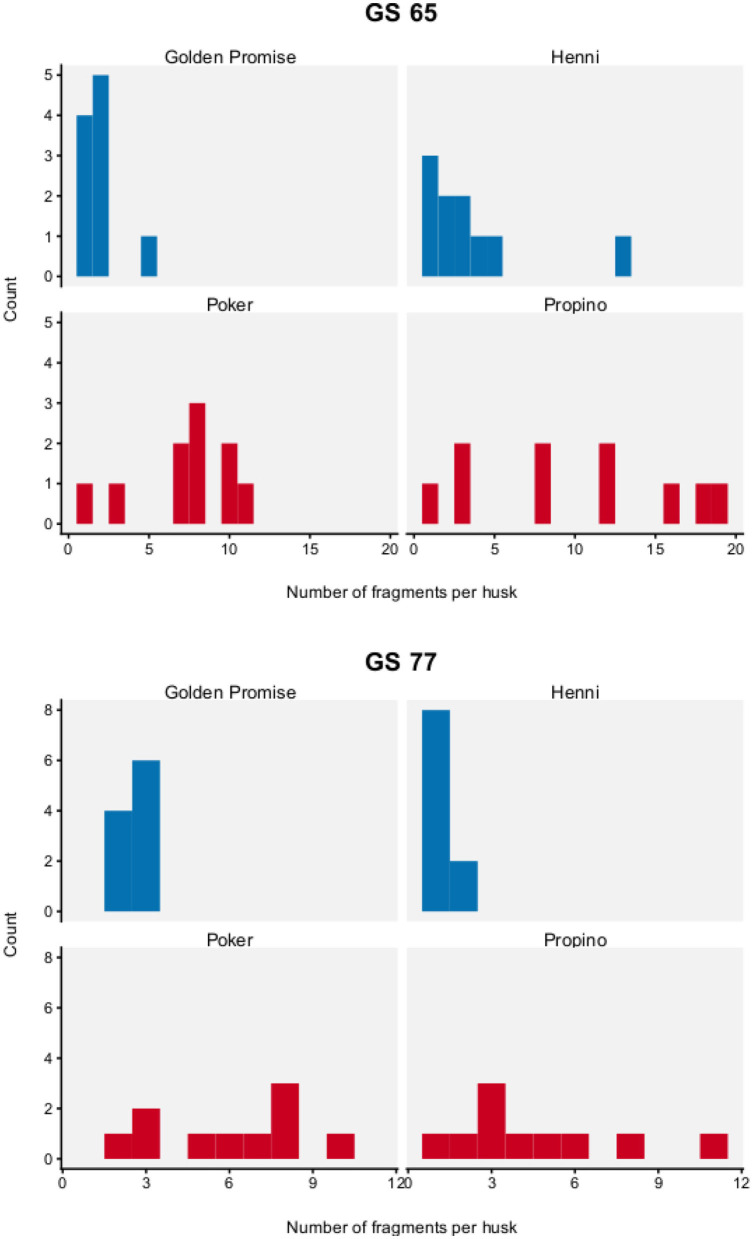
Fragment number. Graph displays a histogram of fragment number. *N* = 10 impact assays per variety × growth stage combination. High risk varieties are shown in red, low risk varieties are shown in blue.

Figure 5 shows the distribution of fragment number by genotype and growth stage. To test for differences between barley varieties, fragment number was modeled with a Poisson distribution, which has one parameter λ. The parameter λ describes how frequently an event occurs in a finite space. In this study, it can be interpreted as the number of fragmentation events that occurred in the lemma.

Fragment number was significantly influenced by genotype and developmental stage. Fragment number was significantly higher in the high risk varieties, compared to the low risk varieties.

**Figure 6:**
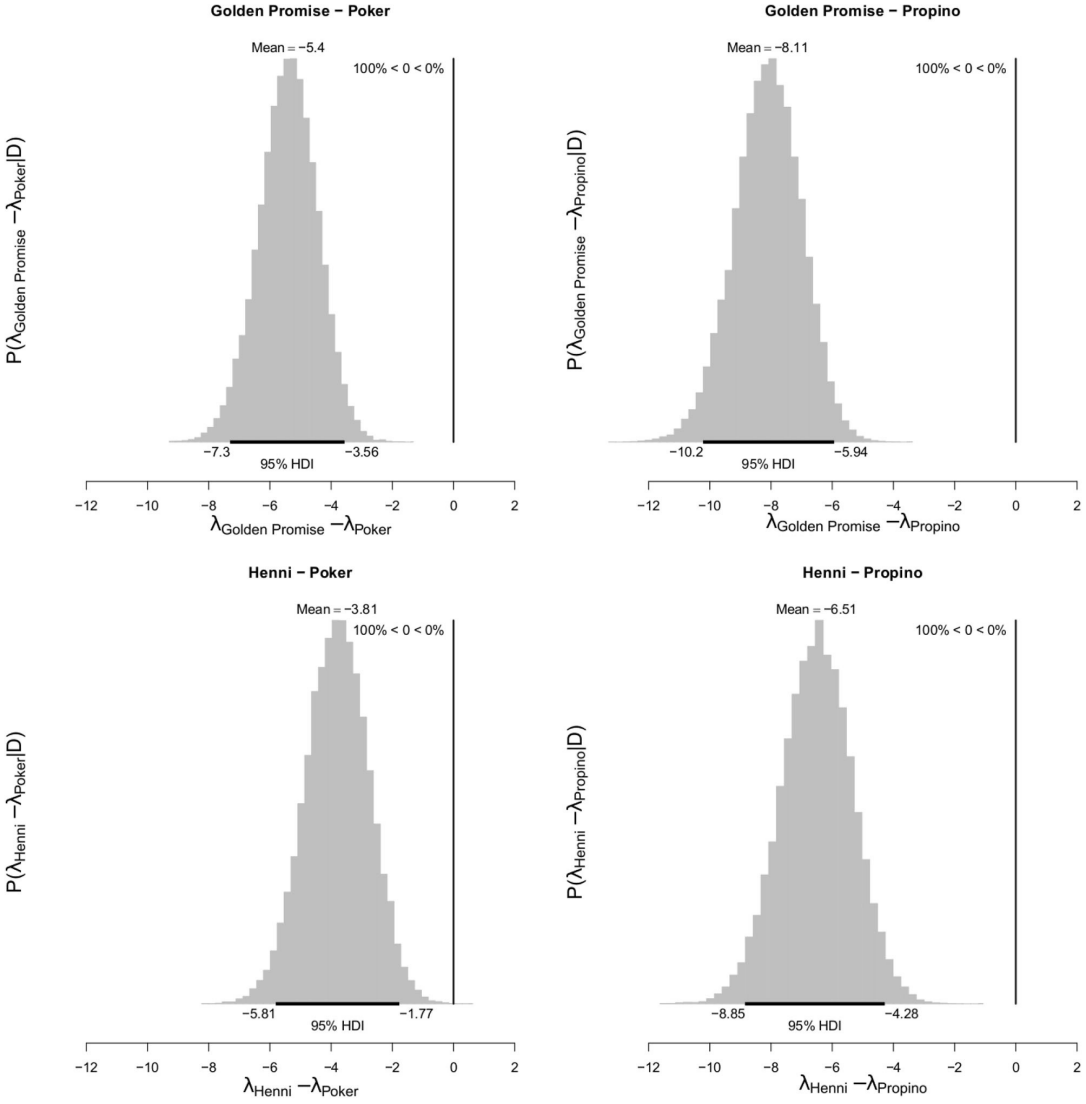
Differences in mean fragment number λ between varieties at GS 65. High risk varieties had higher estimates of λ suggesting that, during impact, more fragments formed. The difference in λ between low risk and high risk varieties were calculated. Graphs show the posterior distribution on the differences between λ, including the mean difference and the 95% HDI of the mean difference. All differences were significantly <0. *N* = 10 impact assays per variety × growth stage combination.

At GS 65, the high risk varieties, Poker and Propino, formed 7.30 fragments (95% HDI of λ = 5.71, 9.04) and 10.00 fragments (95% HDI of λ = 8.08, 11.98) per lemma, respectively. In contrast, the low risk varieties had fewer fragments per lemma: at GS 65, Golden Promise formed 1.90 fragments (95% HDI of λ = 1.10, 2.78) and Henni formed 3.50 fragments (95% HDI of λ = 2.38, 4.68). Figure 6 shows the pairwise differences between varieties.

**Figure 7:**
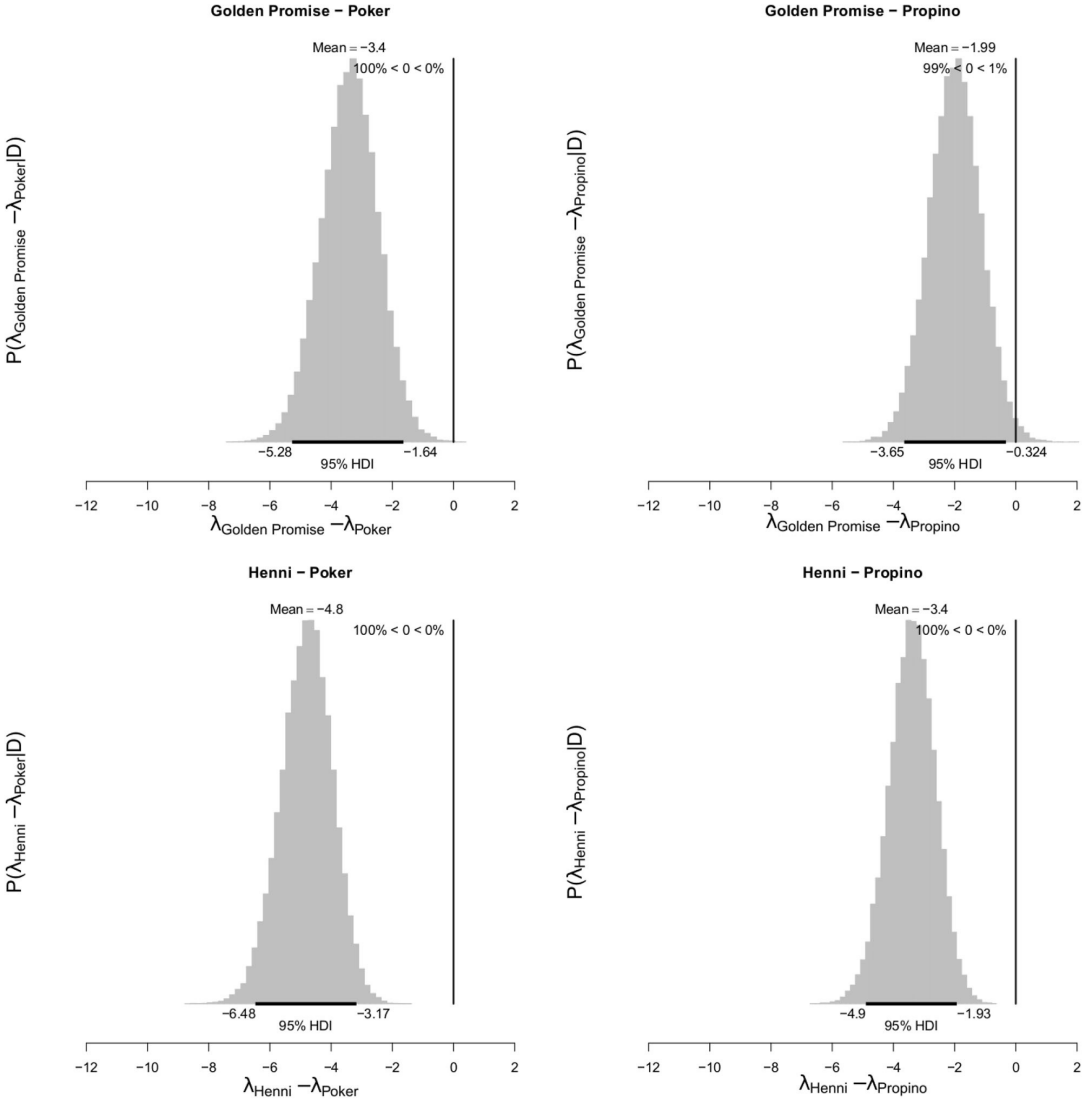
Differences in mean fragment number λ between varieties at GS 77. High risk varieties had higher estimates of λ suggesting that, during impact, more fragments formed. The difference in λ between low risk and high risk varieties were calculated. Graphs show the posterior distribution on the differences between λ, including the mean difference and the 95% HDI of the mean difference. All differences were significantly <0. *N* = 10 impact assays per variety × growth stage combination.

At GS 77, Poker formed 6.00 fragments (95% HDI of λ = 4.53, 7.53) and Propino formed 4.60 fragments (95% HDI of λ = 3.30, 5.92). In contrast, at GS 77, Golden Promise formed 2.60 fragments (995% HDI of λ = 0.64, 3.62) on average and Henni formed 1.20 fragments (95% HDI of λ = 0.56, 1.89) on average. Figure 7 shows the pairwise differences between varieties.

Further information is available in the Supplementary Material, including: fragment number shown by growth stage and variety; the posterior distribution of λ; and the posterior predictive fits.

#### 2.2.2. Fragment Area

**Figure 8:**
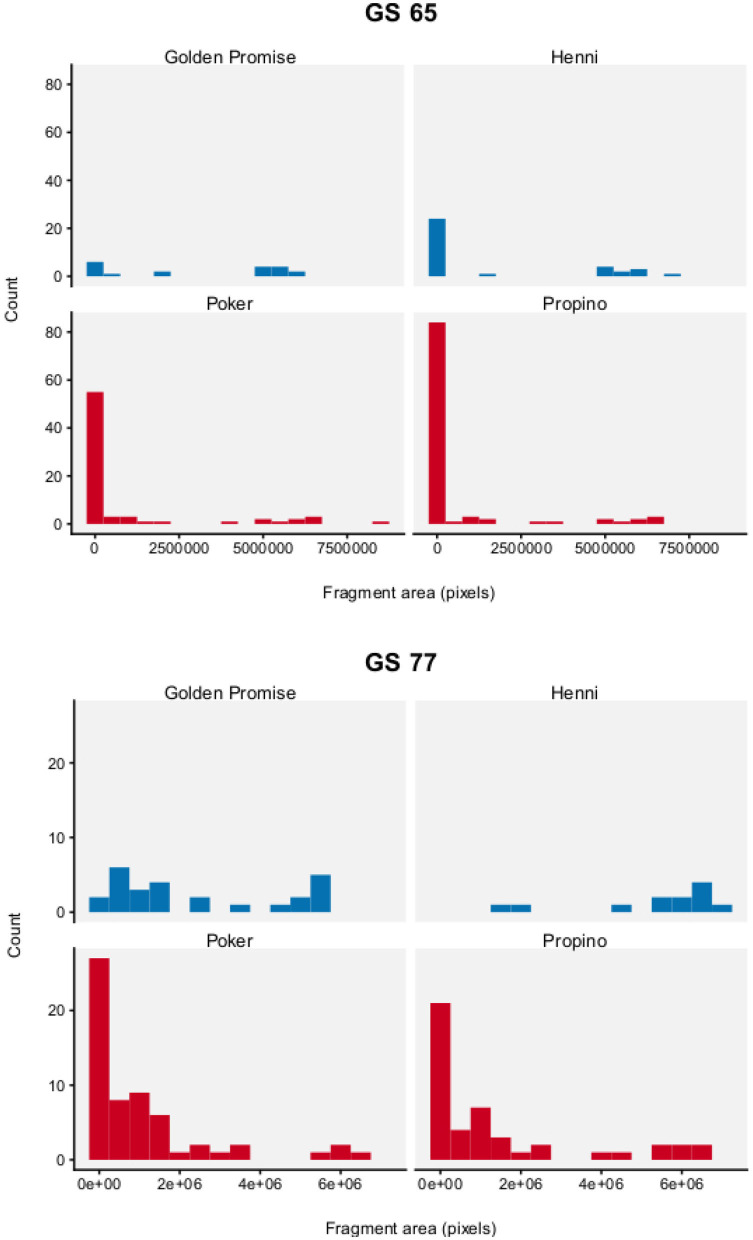
Fragment area. Graph displays a histogram of fragment areas. *N* = 10 impact assays per variety × growth stage combination. High risk varieties are shown in red, low risk varieties are shown in blue.

Figure 8 show the distribution of fragment area by genotype and growth stage. To test for differences between barley varieties, fragment area was modeled with a Gamma distribution. The expectation of the Gamma distribution—estimated from the parameters α and β—can be interpreted as the mean fragment area μ, which was the main parameter of interest.

Fragment area was significantly influenced by genotype and developmental stage. Overall, there is good evidence that fragment area was smaller in the high risk barley varieties, compared to the low risk barley varieties.

**Figure 9:**
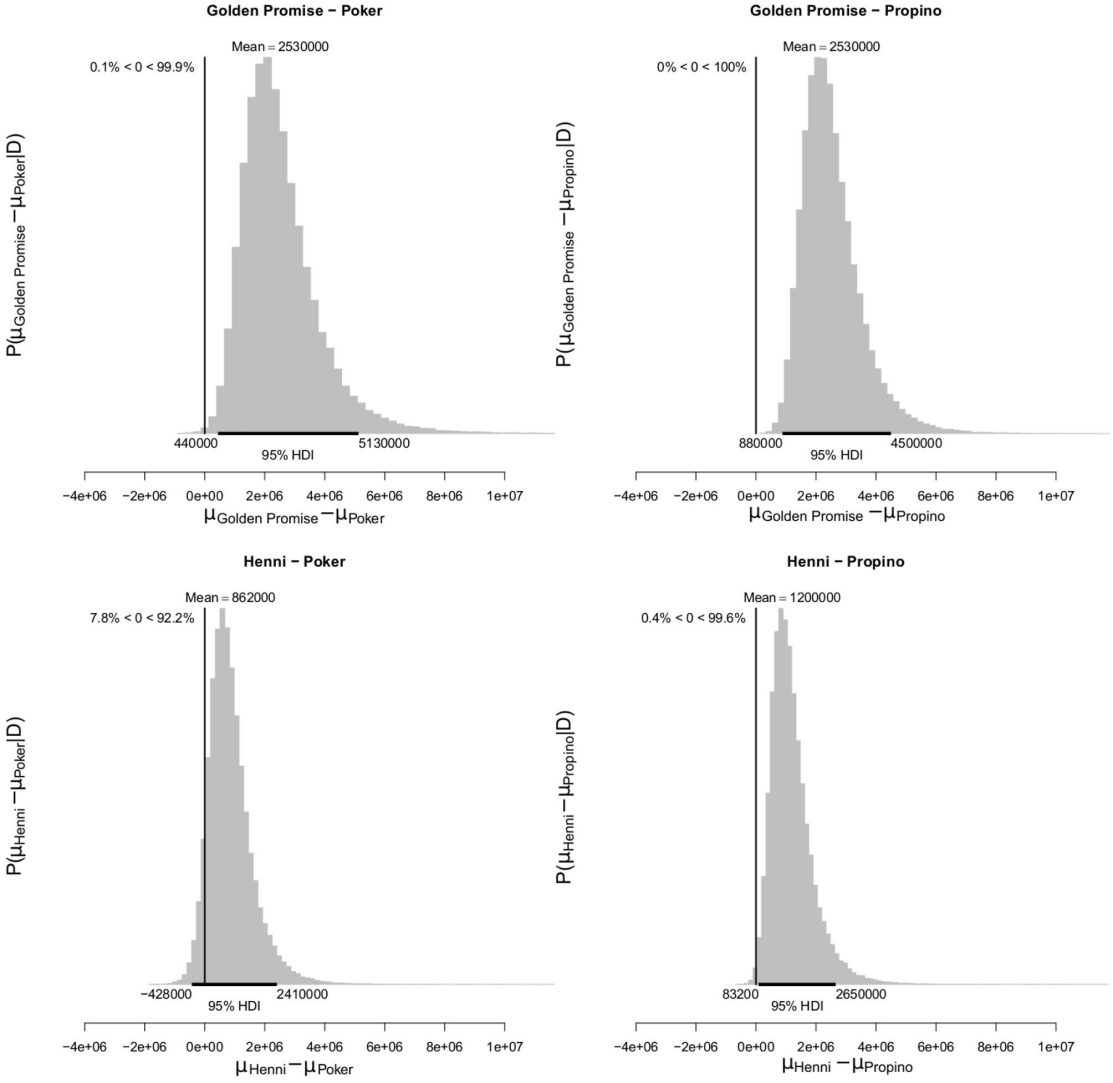
Differences in mean fragment area μ at GS 65. High risk varieties had lower estimates of μ suggesting that, during impact, smaller fragments formed. The differences in μ between low risk and high risk varieties were calculated. Graphs show the posterior distribution on the differences between μ, including the mean difference and the 95% HDI of the mean difference. All differences were >0, with one exception; the difference between Henni and Propino was not significant at the 0.95 level, although the posterior probability of the difference being >0 was 0.92. *N* = 10 impact assays per variety × growth stage combination.

At GS 65, mean fragment area μ was smaller in the high risk varieties, compared to the low risk varieties. Mean fragment area was estimated to be 3.52 × 10^6^ pixels (95% HDI = 1.52, 5.97) in Golden Promise and 1.85 × 10^6^ pixels (95% HDI = 0.75, 3.25) in Henni. The estimate for Poker was 0.98 × 10^6^ pixels (95% HDI = 0.55, 1.51) and for Propino was 0.65 × 10^6^ pixels (95% HDI = 0.41, 0.92). Figure 9 shows the pairwise differences in μ between the low risk and high risk varieties. All differences were significant at the 95% level of confidence, except for the comparison between Henni and Poker. However, there was greater evidence for a difference, than for no difference.

**Figure 10:**
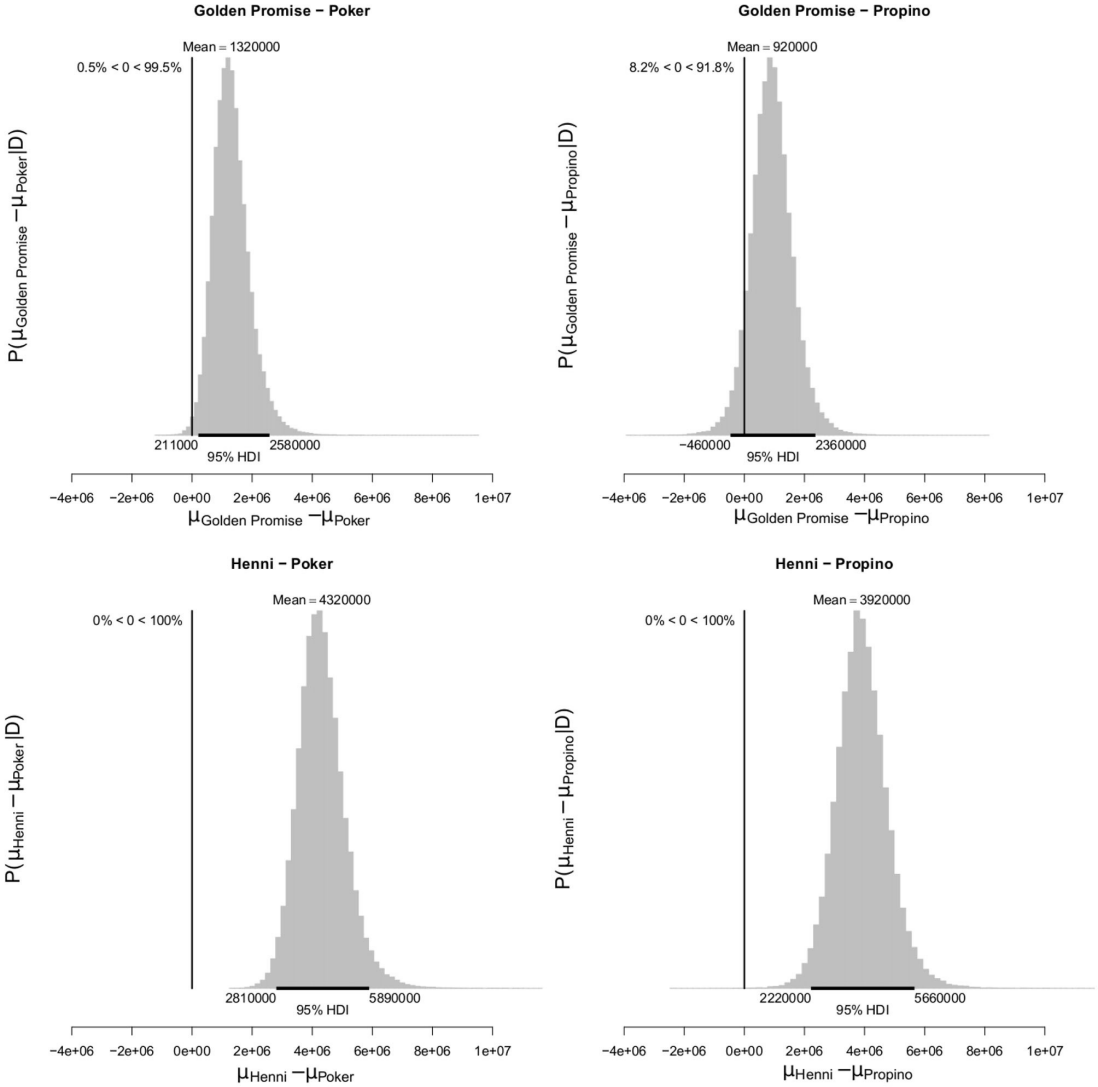
Differences in mean fragment area μ at GS 77. High risk varieties had lower estimates of μ suggesting that, during impact, smaller fragments formed. The differences in μ between low risk and high risk varieties were calculated. Graphs show the posterior distribution on the differences between μ, including the mean difference and the 95% HDI of the mean difference. All differences were >0, with one exception; the difference between Golden Promise and Propino was not significant at the 0.95 level, although the posterior probability of the difference being >0 was 0.94. *N* = 10 impact assays per variety × growth stage combination.

At GS 77, mean fragment area μ was also smaller in the high risk varieties, compared to the low risk varieties. Mean fragment area was estimated to be 2.45 × 10^6^ pixels (95% HDI = 1.49, 3.58) in Golden Promise and 5.45 × 10^6^ pixels (95% HDI = 4.05, 6.97) in Henni. The estimate for Poker was 1.13 × 10^6^ pixels (95% HDI = 0.71, 1.62) and for Propino was 1.53 × 10^6^ pixels (95% HDI = 0.81, 2.38). Figure 10 shows the pairwise differences in μ between the low risk and high risk varieties. All differences were significant at the 95% level of confidence, except for the comparison between Golden Promise and Propino. However, there was greater evidence for a difference, than for no difference.

Further information is available in the Supplementary Material, including: the distribution of fragment areas shown by growth stage and variety; the posterior distributions of α, β, and μ at GS 65 and GS 77; and the posterior predictive fits.

## 3. Discussion

### 3.1. Husk Structure

There is an innate relationship between structure and material properties. Plants adapt their structure to deal with external mechanical stresses (Niklas, 1992, 1999). Several structural features would be expected to influence material properties at the plant organ level, such as the barley lemma or palea (Gibson, 2012; Burgert and Keplinger, 2013; Faisal et al., 2013; Jensen and Fozard, 2015; Brulé et al., 2016; Shah et al., 2017).

#### 3.1.1. Thickness

There can be a very simple relationship between material thickness and material properties. Failure is more likely in a thin material than a thick material, if both materials have the same internal structure and composition. In the thin material, the overall energy per unit volume will always be greater than in the thick material. Therefore, having a thinner husk is generally expected to increase material failure. In this study, lemma thickness was associated with differences in lemma fragmentation between genotypes. The lemma was consistently thinner in the high risk varieties, by up to a factor of 0.61 (95% CI = 0.51, 0.72).

Thin husks have been anecdotally linked to grain skinning in several studies (Roumeliotis et al., 1999; Fox et al., 2006; Fox, 2008), although this is the first time data have been published to support this hypothesis. Thin husks have long been seen as a desirable trait by the malting industry, as they are believed to increase malting performance. Good quality malting barley varieties usually have a thinner husk compared to poor quality malting barley varieties, or feed barley (López-Perea et al., 2012). Several studies have found that husk mass has a negative correlation with malt extract (Collins et al., 1991; Roumeliotis et al., 1999; Roumeliotis and Eglinton, 2007). Malt extract is a key metric for the breeding and selection of malting-specific barleys. It is clear that barley cultivars have faced selection pressure for malt extract. As shown by several studies, malt extract of barley varieties has increased over the last 30–50 years, almost entirely due to genetic improvement (Psota et al., 2009; Laidig et al., 2017). In selecting for high malt extract, it is possible that there has been indirect selection pressure for thin husks. Fox et al. (2006) and Fox (2008) showed that husk thickness was a heritable trait, so selection pressure could result in thin or thick husked genotypes.

Brennan et al. (2017b) found that the severity and frequency of grain skinning was greater in the more modern malting barley varieties, such as Propino, compared to older cultivars, such as Golden Promise. It is plausible that selection pressure for high malt extract has reduced husk thickness in more modern varieties, increasing their grain skinning risk.

Lemma thickness did not explain the differences in fragmentation between growth stages. Growth stage had no significant effect on lemma thickness, whereas the lemma became more resistant to fragmentation between GS 65–77. This was observed across all varieties, so the difference between varieties was maintained. Differences between the growth stages may be explained by changes in lemma composition or lemma density. For example, as they develop, many plants cells develop a thickened, lignified secondary cell wall (Vogel, 2008). This would alter both the density and composition of the lemma, without altering lemma thickness. This would be expected to change the tissue material properties and thus fragmentation behavior. Further work would be needed to unravel the relative contributions of thickness, density and composition to the material properties of the lemma.

#### 3.1.2. Vascular Bundles

There is a well-known relationship between plant vasculature and material properties. The mechanical properties of plant tissues often correlate with vascular tissue (Niklas, 1999; Gibson, 2012). Studies with *Arabidopsis thaliana* have shown that both the presence of vascular bundles (Zhong et al., 1997) and the diameter of vascular bundles (Zhong and Ye, 2004) influence tissue strength. Within the grass family (*Poaceae*), a relationship has been found between vasculature and tissue stiffness in palms (Kuo-Huang et al., 2004; Rüggeberg et al., 2008), reeds (Spatz et al., 1997; Rüggeberg et al., 2009), and bamboo (Wang et al., 2011). Based on these studies, the number, distribution, diameter, and composition of vascular bundles are expected to influence plant material properties.

In the barley husk, the number and distribution of vascular bundles is fixed, so this study has focused solely on the diameter of vascular bundles in the lemma. The diameter of the lemma vascular bundles did not explain the differences in material properties between genotypes. Poker seemed to have a narrower central vascular bundle than the other three varieties; otherwise there were no significant differences between varieties. There were also no significant differences in vascular bundle thickness between growth stages.

There has been one other study which examined the relationship between husk vasculature and grain skinning. Olkku et al. (2005) concluded that grain skinning was more likely in varieties with a thick dorsal vein. This is a surprising result, given what is known about the relationship between vasculature and plant material properties. Unfortunately the authors did not make a quantitative study of these variables, making it difficult to critically evaluate their results.

#### 3.1.3. Cellular Organization

Finally, there are complex relationships between cell organization—cell number, cell size, cell shape—and material properties (Brulé et al., 2016; Shah et al., 2017). Theoretically, increasing cell number means that mechanical stress is shared by a greater number of units. For example, Huang et al. (2001) found that the *Arabidopsis* inflorescence stem was stiffer in the *sturdy* mutant compared to wild-type, thought to result from increased cell numbers in the stem. Theoretically, reducing cell size increases the proportion of cell wall material per unit of tissue. This effectively makes the tissue denser and reduces the mechanical stress per unit volume of tissue. For example, Paul-Victor and Rowe (2010) found that mechanically perturbed *Arabidopsis* plants had significantly increased cell diameters in the supporting interfascicular tissues, significantly reducing stiffness, by a quarter to a third compared to control plants. Cell number and area are usually correlated with tissue thickness and density.

There are also complex relationships between cell type and material properties. Strength and stiffness are greater in the supportive sclerenchymal tissues than parenchymal tissues, likely due to lignification of the thick secondary walls. For example, stiffness (Young's modulus) in parenchymal tissue ranges between 0.0003–0.014 GPa, whereas in vascular cells, it ranges between 10–35 GPa (Niklas and Paolillo, 1997; Köhler and Spatz, 2002; Gibson, 2012; Shah et al., 2017). Thus, the relative proportions of cell types—sclerenchyma to parenchyma—affects mechanical properties, as was found in grasses by Vincent (1982). The relationship between parenchyma and material properties is not straightforward, however. Despite its reduced strength and stiffness, thick parenchymal layers in the pericarp are key for impact resistance in coconuts and pomelos (Seidel et al., 2010; Masselter and Speck, 2011). Due to its structure, parenchyma can absorb a considerable amount of energy (Gibson, 2005, 2012).

In the barley husk, the sclerenchyma forms a stiff, supportive tissue layer just under the outer epidermis. This layer has thicker cell walls, consisting of cellulose, hemicellulose and lignin. In contrast, the parenchyma cells, which are found under the sclerenchyma, have a larger cross-sectional area, surrounded by thin and flexible cell walls. Parenchymal cell walls tend to be rich in cellulose and hemicelluloses. As the grain matures, water is lost from the husk. Parenchyma cells crush together to form a fragmentary layer (Evers and Millar, 2002; see also micrographs in Olkku et al., 2005). As the husk parenchyma are crushed together, the tissue lacks the cellular structure key to impact resistance in other fruit (Seidel et al., 2010; Masselter and Speck, 2011).

In the lemma, sclerenchymal cross-sectional cell area did not explain the differences in fragmentation between genotypes. Although there were significant differences in cell area between genotypes, this did not correlate with fragmentation or grain skinning risk. There were also no differences in cell area between growth stages.

### 3.2. Husk Material Properties

#### 3.2.1. Husk Fragmentation Is Influenced by Genotype, Growth Stage, and Structure

The results indicate a relationship between reduced lemma thickness and increased fragmentation. Two barley varieties, Propino and Poker, had significant thinner lemma tissue at both growth stages. Consequently, they were highly susceptible to husk fragmentation, producing the highest number of lemma fragments and the smallest lemma fragments. These two varieties have consistently had an extremely high risk of grain skinning in previous work (Brennan et al., 2017b, 2019; Grant, 2019).

The inverse relationship was found in the other two barley varieties, Henni and Golden Promise. These varieties had significantly thicker lemma tissue at both growth stages. They were highly resistant to husk fragmentation, producing the fewest lemma fragments, with the largest areas. In previous work, they have consistently had an extremely low risk of grain skinning (Brennan et al., 2017b, 2019; Grant, 2019).

The difference between barley varieties was evident at anthesis (GS 65), suggesting that these differences are established during floral development. During grain development, the material properties of the lemma appear to change, leading to an overall lower number fragments at GS 77 across all varieties. However, the clear difference in fragmentation between the low risk and high risk barley varieties was maintained. As discussed previously, differences between the growth stages was not associated with lemma thickness, but may be explained by changes in lemma composition or lemma density.

#### 3.2.2. Evaluating Methods Used to Assess Husk Fragmentation

Many traditional mechanical tests have been adapted to allow the material properties of plant tissues to be assessed (reviewed in Shah et al., 2017). Choosing an appropriate test is important, as biological materials are highly anisotropic, so the values obtained when measuring their material properties depend on the direction of load. The material properties of plant tissues have been measured using tensile (Cavalier et al., 2008; Abasolo et al., 2009), compressive (Wright et al., 2005; Tavakoli et al., 2009), and flexural tests (Lemloh et al., 2014; Robertson et al., 2015). However impact, such as the impact experienced during threshing and which causes grain skinning, presents a unique challenge to materials. Currently in plant biology, there are very few studies into impact resistance. In a literature review, the only examples found were resistance to rockfall in trees (Huang et al., 2018; Olmedo et al., 2018) and resistance to impact in falling fruit (Seidel et al., 2010; Masselter and Speck, 2011). No studies were found on the fragmentation of plant tissues in response to impact; this makes grain skinning a unique case study.

Inspiration was instead drawn from materials science, where there have been many studies into the impact-induced fragmentation of rock, soil and ceramics (Shockey et al., 1974; Santurbano and Fairhurst, 1991; Perfect, 1997; Chau et al., 2000; Salman et al., 2004; Gorham and Salman, 2005; Sanchidrián et al., 2012, 2014; Ghanbarian and Daigle, 2015; Paluszny et al., 2016; Xu et al., 2016). Their methods were adapted to develop a simple impact assay to test for differences in husk fragmentation between genotypes. The impact assay was designed to test the material properties of the husk in isolation from the grain system. If a husk were susceptible to impact, it would be expected to form (1) more fragments and (2) a higher proportion of small fragments than a husk resistant to impact.

Lemma fragment number was modeled using a Poisson distribution. Future work may wish to develop this model further, as certain assumptions made by the Poisson distribution may be problematic in the context of fragmentation. The Poisson distribution assumes that events are independent, however, the formation of one fragment may increase the likelihood of further fragments forming. It also assumes that events cannot occur at the same instant, however, it is possible that multiple fragments may form simultaneously. Despite this, overall, the posterior predictive fits suggested that the data could be accurately modeled by the Poisson model.

Lemma fragment area was modeled using a Gamma distribution. Historically, the Weibull distribution (Weibull, 1951) has been used to model fracture mechanics in brittle materials, including the distribution of fragment sizes (see, for example, Paluszny et al., 2016 and Krifa, 2009). For this study, the Weibull distribution was tested prior to data analysis, however, the Gamma distribution produced the best posterior predictive fits for the lemma fragmentation data. This is consistent with comparative reviews, which found that other distributions, including Gamma, may describe fragmentation better than Weibull (Lu et al., 2002; Basu et al., 2009).

The distribution of fragment areas may have a subtle bimodal distribution, probably introduced by the underlying biomechanics of fragmentation. During impact, the first fragments generally formed from the outer edges of the lemma and were medium-sized. As impact continued, these medium fragments sub-divided more rapidly, forming progressively smaller fragments. Thus, the impact assay tended to result in one or two large fragments (parent fragments) and many small fragments (child fragments). As a result, the Gamma model may underestimate the proportion of large parent fragments. This could be accounted for in a number of ways. Firstly, a bimodal Gamma model could be fitted to the parent and child fragment sub-populations. Bimodal models have been commonly used in fragment size analyses (Krifa, 2009; Sanchidrián et al., 2012, 2014). To illustrate this suggestion, a bimodal model is fully described in section 5.5.3 of the Supplementary Material and applied to the lemma fragmentation data. However, this technique was not used for this study, as there was insufficient experimental power to estimate the Supplementary parameters required. Nevertheless, it demonstrates how the current analysis could be extended in future work. Secondly, fragmentation is a fractal process. The hierarchical relationship between a parent object and its progeny fragments can be modeled using a fractal distribution (Turcotte, 1986; Perfect, 1997; Ghanbarian and Daigle, 2015; Xu et al., 2016). Future work could therefore attempt to model husk fragmentation using a fractal distribution.

#### 3.2.3. Relating Husk Fragmentation to Husk Material Properties

The impact assay was sufficient to demonstrate that lemma fragmentation was significantly affected by genotype and developmental stage. However, this is not a formal measure of material properties and further experiments are required to offer a truly biomechanical explanation for these observations. Fracture mechanics suggests that three specific material properties—strength, stiffness, and toughness—influence fragmentation (Zhuang and Liu, 2014b; Chang, 2014; Zhuang and Liu, 2014a). This leads to a clear hypothesis for future research: do some barley varieties have lemmas with altered strength, stiffness, or toughness, leading to an increased risk of impact-induced fragmentation and thus grain skinning.

Although simple, this hypothesis may prove challenging to test in practice. Material properties are more straightforward to measure in many engineering materials, such as steel; these properties are insensitive to sample size in homogeneous materials. However, plant materials behave very differently, due to their layered construction: with cell wall polymers, cells, tissues, and organs. This means that material properties can be measured at different levels (Gibson, 2012; Burgert and Keplinger, 2013; Faisal et al., 2013; Jensen and Fozard, 2015; Brulé et al., 2016; Shah et al., 2017). Should lemma material properties be measured at the level of the organ or the cell?

Material properties for overall lemma structure could theoretically be measured in a universal testing machine (UTM). Plant tissue is difficult to use with UTMs, although it has been achieved successfully (Bidhendi and Geitmann, 2018; Stubbs et al., 2018). Optimizing existing protocols to deal with barley husks will be a challenging project, given their small size, complex morphology and delicate tissue.

Alternatively, at a sub-cellular level, the material properties of lemma cell walls could be estimated using techniques, such as atomic force microscopy (AFM) or cellular force microscopy (CFM), as described in Burgert and Keplinger (2013) and Vogler et al. (2015). However, due to the small surface areas tested by these technologies, any measurements would reflect very localized material properties. Material properties will depend heavily on the tissue layer tested. For example, parenchymal cell walls almost certainly have different material properties to sclerenchymal cell walls, due to differences in cell wall structure and composition (this is discussed further, in a later section). AFM or CFM would have to be used and interpreted with care.

### 3.3. Understanding Grain Skinning

This paper described two possible two mechanisms which could result in grain skinning: adhesion failure or substrate failure. Distinguishing which mechanisms occur in barley is important. From a biomechanical perspective, adhesion failure and substrate failure are very different mechanisms requiring different breeding strategies. For example, in adhesion failure, the material properties of the adhesive itself would be very important, so breeding strategies might focus on the structure and composition of the pericarp. However, in substrate failure, the material properties of the husk or caryopsis tissue would form a key part of any breeding strategy.

This study challenges the current assumption that grain skinning is caused by adhesion failure alone. Instead, differences in the material properties of the husk tissue were sufficient to explain differences in grain skinning between genotypes in this study. This does exclude adhesion failure from playing a role in grain skinning, but the relative importance of adhesion failure should be re-evaluated concurrently with substrate failure.

There is evidence for both mechanisms—adhesion and substrate failure—in previous literature. Previously published micrographs appear to show the husk detaching from the caryopsis along the cementing layer (Gaines et al., 1985; Hoad et al., 2016), which is consistent with adhesion failure. Other micrographs show fractured husk parenchyma (Okoro et al., 2017, personal communication; Olkku et al., 2005), which would be consistent with substrate failure. Several publications also have photographs or describe skinned grains with a damaged or fragmented husk, also indicative of failure in the husk material (Harlan and Martini, 1936; Reinbergs and Huntley, 1957; Fisher, 1970; Rajasekaran et al., 2004; Olkku et al., 2005; Brennan et al., 2019).

These results also have important implications for the future study of husk adhesion in barley. Research into the development of husk adhesion has used grain skinning as a proxy measurement of adhesion quality (Hoad et al., 2016; Brennan et al., 2017a,b, 2019). However, the present study showed that grain skinning due to adhesion failure may be conflated with grain skinning due to husk material failure. The latter mechanism is unlikely to be an indicator of adhesion quality. Future studies should therefore avoid using grain skinning as a proxy measure of adhesion.

## 4. Conclusions

This paper explored the link between plant development, plant structure, plant material properties and the effect this has on barley used in the malting industry.

The lemma, a barley husk organ, was examined during plant development. This study concludes that lemma structure is set during floral development, as no changes in thickness, vascular bundle diameter or sclerenchymal cell area were observed after anthesis (GS 65). It was hypothesized that husk structure determines its material properties, with fragmentation behavior used as a proxy measure for material properties in this study. Lemma thickness was clearly associated with changes in fragmentation behavior, although there was no clear association between vein diameter or sclerenchymal cell area and fragmentation behavior. Specifically, having a thinner lemma was associated with an increase the risk of lemma fragmentation.

This result has important implications for the use of barley in the malting industry. A barley grain must be able to withstand mechanical stress, such as that experienced mechanized harvesting and processing. Two barley varieties tested in this study, Poker and Propino, had significantly thinner lemma tissue, so the lemma showed higher levels of fragmentation in response to mechanical stress. It is therefore expected that both varieties would suffer husk damage—possibly leading to husk loss—during industrial processing and it is no surprise that other publications have found that both varieties have a high risk of grain skinning, an industry measure of husk loss (Brennan et al., 2017b, 2019; Grant, 2019). Likewise, the other barley varieties tested, Henni and Golden Promise, had significantly thicker lemma tissue, so the lemma showed lower levels of fragmentation in response to mechanical stress. It is therefore expected that both varieties would be more resistant to husk damage and husk loss during industrial processing. This prediction which is consistent with other publications, who have found that these varieties have a low risk of grain skinning (Brennan et al., 2017b, 2019; Grant, 2019). Consequently, we hypothesize that a thick husk protects the grain from mechanical stress and therefore reduces grain skinning risk.

These results raise important questions about breeding barley varieties for malting quality. Whilst a thin husk is considered to be beneficial for some aspects of malting quality—for example it is associated with increased hot water extract (Collins et al., 1991; Roumeliotis et al., 1999; Roumeliotis and Eglinton, 2007)—it may increase the risk of grain skinning, which is detrimental for malting quality. Brennan et al. (2017b) found that the severity and frequency of grain skinning was greater in the more recent malting barley varieties, such as Propino. The results from this study suggest that continued breeding for a thin husk and a high hot water extract may exacerbate this trend.

## 5. Materials and Methods

### 5.1. Plant Material

Malting barley varieties were selected based on previous work on grain skinning. To maximize the number of replicates in this study, the number of varieties selected was kept low. The four varieties chosen—Henni, Golden Promise, Poker, and Propino—have repeatedly shown extreme grain skinning phenotypes across a number of experiments (Brennan et al., 2017b, 2019; Grant, 2019). All varieties are part of the IMPROMALT collection maintained by the James Hutton Institute. Information on variety pedigree, release date and skinning phenotype is shown in Table 1.

**Table 1:** Information on the four extreme malting barley varieties used in this study.

**Variety**	**Release date**	**Parents**	**Breeder**	**Grain skinning risk**
Golden Promise	1968	Gamma ray mutant (Maythorpe)	Milns Seeds, UK	Extreme low
Henni	1994	Baronesse ×84160/1/3/3	Noordsaat, DE	Extreme low
Poker	2003	SJ 96-1441 × Colston	Syngenta, UK	Extreme high
Propino	2007	Quench × NFC Tipple	Syngenta, UK	Extreme high

### 5.2. Plant Growth Conditions

Malting barley varieties were grown in a research greenhouse located at Scotland's Rural College (SRUC) in the UK (55° 55′17.8″ N, 3° 10′44.1″ W). The greenhouse compartment had a floor area of 9.5 × 5.5 m (52.25 m^2^) and was bounded by brick wall 0.75 m high. The compartment was 2.5 m high, with a peaked roof. It had a bench area of 1 × 6 m (6 m^2^), elevated 0.85 m from the floor. The greenhouse compartment was oriented along the North-South axis. The compartment had two internal walls (to the North and East) and two external walls (to the South and West). The East wall was bounded by identical, but differently controlled greenhouse compartments. The North wall was bounded by a central corridor between the different compartments and was constructed from brick. The rest of the compartment was constructed from glass. The compartment had two thermostatically controlled vents, one located in the window of the South wall (4.4 × 0.5 m in area) and one located in the roof: (4.4 × 2 m in area). Both vents were covered in an insect screen. The compartment did not have a shade screen.

The greenhouse was equipped with a supplementary lighting system (Mercury vapor lamps, Hortilux Schreder, 400 W/230 V) capable of providing a minimum level of photosynthetically active radiation (PAR) flux of 150 μmol m^−2^ s^−1^ at the canopy level. A 16 h photoperiod was imposed by operating the supplementary lighting system when no sunlight was available. Solar radiation was monitored; data from a local weather station shows that the average solar radiation during the experiment was 0.17 kW m^−2^ (σ = 0.28, see Supplementary Material). However, the supplementary lighting system was turned on when solar radiation dropped below 0.1185 kW m^−2^ (15 K lux).

The greenhouse compartment heating system was set to maintain a minimum temperature of 15°C during the day and 10°C during the night. Air temperature in the compartment was recorded every 8 min over a 24 h period using a Delta-T Data Logger (Cambridge, UK). The mean air temperature over the experiment was 16.19°C (σ= 2.46°C, see Supplementary Material). Humidity in the greenhouse compartment was not controlled, however it was also monitored every 8 min over a 24 h period using a Delta-T Data Logger (Cambridge, UK). During the experiment, mean relative humidity was 50.77% (σ= 7.65%, see Supplementary Material). All sensors were calibrated annually according to manufacturers guidance.

Plants were grown between January and May 2018. Plant growth was assessed and reported using the decimal code for cereal growth stages (GS) as described in (Tottman, 1987). Plastic pots (5 L) were prepared with Levington John Innes No. 2 compost and 5 seeds were sown into each pot at a depth of 2.5 cm. Pots checked daily and were watered when the soil surface was dry. Pots were not given any additional nutrients. Pots were aligned in a 4 × 10 configuration. Until GS 59, pot density was 40 in a 2.6 × 1 m area. Pots were rearranged just before flowering (GS 59) to allow sampling to take place with minimal disturbance. Therefore, from GS 59 onwards, pot density was 40 in 3.5 × 1 m. Flowering occurred between 15th March and 11th April 2018 and samples were collected between 16th March and 21st April 2018.

### 5.3. Replication and Sampling

For each variety, 5 plants ×10 pots were sown, leading to a total of 50 plants per variety. Germination was good for all varieties apart from Golden Promise, where only 60% of seeds germinated. Unfortunately, this means that there were lower numbers of replicates for Golden Promise for the analysis of husk structure.

The main ear of each plant was given a unique identity tag during booting (GS 41). After tagging, the ear was assigned to a random sampling group. Supplementary Figure 12 gives an overview of the sampling scheme used in this experiment. Each pot had 1 ear sampled on the day of anthesis (GS 65) and 1 ear sampled during grain filling (GS 77). At both growth stages, 3 grains were taken from this central region to examine husk structure. A further 3 grains were also taken from this central region to examine husk material properties. At each growth stage, each variety had 30 grains from 10 ears (18 grains across 6 ears for Golden Promise) sampled to assess husk structure and 30 grains from 10 ears (18 grains across 6 ears for Golden Promise) sampled to assess husk material properties.

The results for this study are only from one harvest, but this balanced by a high level of replication and the use of four different varieties, allowing a breadth in trait expression.

### 5.4. Husk Structure

Husk structure was assessed at GS 65 and GS 77, in order to examine the link between lemma structure and material properties.

#### 5.4.1. Microscopy

During sampling, the grain was removed from the awn and stored at −20° C. When grains were removed from storage, the lemma and palea were gently dissected from the caryopsis and kept on ice until ready for imaging. A 1 mm section was cut from the center of each lemma, along the transverse axis, using a sharp scalpel. Sections were stained with 1% (w/v) toluidine blue O for 30 s, washed with distilled water and placed on a slide. Slides were analyzed using a microscope (Model BX53F, Olympus, Tokyo, Japan) at ×10 magnification to measure lemma thickness and lemma vascular bundle diameter, or ×20 magnification to measure the cross-sectional area of the schlerenchymal cells in the lemma. Images were captured using a Canon EOS 60D digital SLR camera (Canon, Tokyo, Japan).

#### 5.4.2. Image Analysis

Measurements were taken from the microscopy images using ImageJ (Schindelin et al., 2012). Lemma thickness was measured in the central region, in the mid region and in the lateral region of the lemma. In the lemma, the central measurements were taken between the center vein and mid vein, the mid measurements were taken between the mid vein and lateral vein, and the lateral measurements were taken between the lateral vein and the distal edge of the lemma. Thickness was very variable. It was important to choose regions to measure in a fair and consistent way across samples. Therefore, thickness in each region was measured at the maximum visible point and the minimum visible point. This gave an upper and lower bound for thickness.

The lemma has five vascular bundles: one central vein, two mid veins and two lateral veins. The diameter was measured for one of each vein type.

The cross-sectional area of sclerenchymal cells were measured for the mid and lateral region of the lemma only. It was also not possible to measure the cross-sectional area of cells in the central region of the lemma because cells appeared to have thick, lignified cell walls, meaning that the cell lumens were not clearly visible. Cell area was highly variable. The area of the largest visible sclerenchymal cells were measured and the measurements of the five largest cells were used. This meant that cells were chosen in a consistent way across samples. In both husk organs, the parenchymal cell layer is crushed together. This meant is was also not possible to measure cross-sectional area for the parenchymal cells or to measure the proportion of sclerenchyma to parenchyma accurately. In future work, this could be achieved by fixing and embedding husks, prior to sectioning.

#### 5.4.3. Data Analysis

All structural features were assumed to follow a normal distribution. Analysis of variance tests (ANOVAs) (α = 0.05) were therefore used to determine if genotype and growth stage had a significant effect on each structural feature. *Post-hoc* Tukey's HSD tests (α = 0.05) were used summarize the mean pairwise differences (with a 95% CI) between genotypes and growth stages. Data analysis was carried out in R (R Core Team, 2018).

### 5.5. Husk Material Properties

Husk material properties—specifically, resistance to fragmentation—were assayed during grain development. In order to test the material properties of the husk in isolation, the husk must be removed from the caryopsis. However, this is difficult from GS 83 and impossible after GS 85. Therefore, the material properties of the husk were tested at GS 77, as this is the latest time-point at which the husk can be removed from the caryopsis without introducing structural flaws. Husk fragmentation was also assayed at anthesis (GS 65) to establish whether differences arose during floral development or during grain development.

#### 5.5.1. Impact Assay

During sampling, the lemma and palea were very gently removed from the caryopsis. Care was taken not to introduce any cracks during sampling, as this could introduce a confounding artifact. If cracks were introduced, the grain was discarded and a new grain selected from the central region of the ear. The lemma and palea were stored at −20°C until the impact assay was carried out. It is worth noting that freezing and thawing the husk tissue may alter the material properties. However, all samples received the same treatment, so it is still possible to compare between genotypes and growth stages.

Husks were removed from storage, each individual lemma was placed into a pre-chilled 2 ml Eppendorf tube and kept on ice. A digital dry block heater (AccuBlock^TM^, Labnet International, Inc., Edison, USA) was pre-warmed to 25°C. Lemmas were allowed to defrost for 20 min at 25° C. This reduced the moisture content from 54 to 49% on average. This step was important to allow fragmentation to take place, otherwise the high moisture content meant that the lemma tissue was flexible and therefore naturally resistant to fragmentation. One ceramic ball bearing (6 mm diameter, 0.6725 g in mass) was added to each Eppendorf. The Eppendorfs were placed in a TissueLyser LT (Qiagen, Venlo, Netherlands) and oscillated at 50 Hz for 10 s. The samples were then returned and kept on ice until imaging was complete, to prevent the fragments from shrinking due to water loss. Fragments were analyzed using a dissecting microscope (Model DZ5040, Euromex, Arnhem, Netherlands) at ×1 magnification. Images were captured using a Canon EOS 60D digital SLR camera (Canon, Tokyo, Japan) and processed using ImageJ (Schindelin et al., 2012). After each image was processed to a binary image, the built-in particle analysis module was used to calculate both fragment number and fragment area. The minimum threshold was set to 500 pixels; this was sufficient to measure the smallest lemma fragments whilst eliminating noise.

#### 5.5.2. Data Analysis

Fragment number was analyzed using Bayesian inference. During impact, each individual lemma formed a number of fragments. If no fragmentation event took place and the lemma remained in one piece, then this was treated as 1 fragment. If one fragmentation event took place, then the lemma formed two fragments, and so on. The number of fragments formed from each individual lemma *Y* was assumed to follow a Poisson distribution. The Poisson distribution is used to describe the number of times an event occurs in a defined interval of time or space. The Poisson distribution is described by the rate parameter λ: the mean number of events that occur in an interval of time or space. An estimate and a 95% highest density interval (HDI) for λ was estimated from the posterior distribution of λ. The posterior distribution was calculated using a vague prior, as follows:

(1) $$\begin{array}{r} y_{i}\sim Poisson\left(\lambda_{variety}\right)\\ \lambda_{variety}\sim Gamma\left(0.001,0.001\right) \end{array}$$

The estimates of λ were compared between varieties. Varieties with significantly higher estimates of λ formed significantly more fragments and were therefore considered to be less resistant to impact. Significance was assessed using the 95% HDIs.

Posterior predictive checks are presented in the Supplementary Material. These show the most likely posterior distributions of λ superimposed over the original data. They suggest that the Poisson model was a reasonable fit for the data.

Fragment area was also analyzed using Bayesian inference. Lemmas formed a range of fragments of varying sizes during the impact assay so the Gamma distribution was chosen to model the distribution of fragment areas *Y*. The Gamma distribution belongs to the exponential family of distributions and has a shape parameter α and rate parameter β. An estimate and a 95% HDI for α and β were estimated from their posterior distributions. The posterior distributions were calculated using vague priors, as follows:

(2) $$\begin{array}{r} y_{i}\;\sim \;Gamma\;(\alpha_{variety},\beta_{variety})\\ \alpha_{variety}\;\sim \;Gamma\;(0.001,0.001)\\ \beta_{variety}\;\sim \;Gamma\;(0.001,0.001) \end{array}$$

The shape parameter α determines the shape of the Gamma distribution. Distributions with smaller values of α have a stronger skew toward zero and therefore a higher probability of producing smaller fragments. The rate parameter β determines the scale of the Gamma distribution. The value of β is linked to the largest fragment observed, in all cases here, the area of the intact barley lemma.

The expectation of the Gamma distribution is calculated from the shape and rate parameters:

(3) $$\begin{array}{r} 𝔼\;(Y)=\frac{\alpha_{variety}}{\beta_{variety}}=\mu_{variety} \end{array}$$

The expectation of the Gamma distribution can be interpreted as the mean fragment area. An estimate of the expectation, referred to here as μ_*variety*_ was therefore the main parameter of interest. Estimates of μ_*variety*_ were compared between varieties. Varieties with significantly lower estimates of μ_*variety*_ were likely to form smaller fragments and were thus considered to be less resistant to impact. Significance was assessed using 95% HDIs.

Posterior predictive checks are presented in the Supplementary Material. These show the likely posterior distributions of μ_*variety*_ superimposed over the original data. This suggested that the Gamma model was a reasonable fit for the data, but tended to underestimate the number of large fragments, especially in the low risk varieties. This suggests a more complex model may be needed to describe husk fragmentation.

All posterior distributions were estimated using the Metropolis-Hastings Markov Chain Monte Carlo (MCMC) algorithm, implemented using a Gibbs Sampler (Kruschke, 2014; Kruschke and Liddell, 2018) with the *rjags* package (Plummer, 2016) in R (R Core Team, 2018). To estimate each posterior distribution, three chains were run. Each chain had 10000 iterations for adaptation and burn-in which were discarded. This was followed by 50,000 iterations for the Poisson models or 100,000 iterations for the Gamma models. The parameter space was well-explored by all MCMC chains. The shrink factor (Gelman-Rubin statistic) was below 1.1, indicating chain convergence. The Monte Carlo standard error (MCSE) was generally close to 0, indicating that across the chains, the parameter estimates were similar and precise. Generally, effective sample size was high and auto-correlation was low. The only exception was modeling fragment area for Henni at GS 77, which was highly resistant to fragmentation, so formed few fragments. This meant there was a small number of data points, which caused high levels of auto-correlation. Consequently, the 95% HDIs were wide for this variety, but there were no indications that the parameter estimate was inaccurate.

## Data Availability Statement

The datasets presented in this study can be found in online repositories. The names of the repository/repositories and accession number(s) can be found at Figshare: doi: 10.6084/m9.figshare.11637792; 10.6084/m9.figshare.11637768; 10.6084/m9.figshare.11637777; 10.6084/m9.figshare.11637744; 10.6084/m9.figshare.11637765; 10.6084/m9.figshare.11637780; 10.6084/m9.figshare.11637783.

## Author Contributions

KG conceived and designed the study, conducted the experiment, collected the data, performed the analysis, and drafted the manuscript. MB and SH supervised the project and contributed to the manuscript. All authors have read and approved the manuscript, and agreed to be accountable for the content of the work.

## Conflict of Interest

The authors declare that the research was conducted in the absence of any commercial or financial relationships that could be construed as a potential conflict of interest.

## Supplementary Material

Supplementary figures and tables for publication, available on Figshare (doi: 10.6084/m9.figshare.11637792), including: plant growth conditions; experiment sampling scheme; posterior predictive checks for lemma fragment number and area models; posterior distributions for the parameters (α, β, μ) used for modeling lemma fragment area; supplementary methods and results for using a bimodal model for lemma fragment area; statistical tests for differences in lemma structure.

Supplementary File 1 Supplementary_material.jpeg. Additional figures and tables for publication, including: plant growth conditions; experiment sampling scheme; posterior predictive checks for lemma fragment number and area models; posterior distributions for the parameters (α, β, μ) used for modeling lemma fragment area; supplementary methods and results for using a bimodal model for lemma fragment area; statistical tests for differences in lemma structure. File available on Figshare, doi: 10.6084/m9.figshare.11637792.

Supplementary File 2 DATA_Husk_Fragmentation_GS65.csv. Data on barley lemma fragmentation at GS 65, including fragment area and fragment number. File available on Figshare, doi: 10.6084/m9.figshare.11637768.

Supplementary File 3 DATA_Husk_Fragmentation_GS77.csv. Data on barley lemma fragmentation at GS 77, including fragment area and fragment number. File available on Figshare, doi: 10.6084/m9.figshare.11637777.

Supplementary File 4 DATA_Husk_Structure_GS65.csv. Data on barley lemma structure at GS 65, including lemma thickness, vascular bundle diameter and sclerenchymal cell area. File available on Figshare, doi: 10.6084/m9.figshare.11637744.

Supplementary File 5 DATA_Husk_Structure_GS77.csv. Data on barley lemma structure at GS 77, including lemma thickness, vascular bundle diameter and sclerenchymal cell area. File available on Figshare, doi: 10.6084/m9.figshare.11637765.

Supplementary File 6 ANALYSIS_Husk_Fragmentation.R. R code used to analyse the data, including the Bayesian models, and generate the figures or tables. File available on Figshare, doi: 10.6084/m9.figshare.11637780.

Supplementary File 7 ANALYSIS_Husk_Structure.R. R code used to analyse the data and generate the figures or tables. File available on Figshare, doi: 10.6084/m9.figshare.11637783.

## References

[B1] Abasolo W. Eder M. Yamauchi K. Obel N. Reinecke A. Neumetzler L.. (2009). Pectin may hinder the unfolding of xyloglucan chains during cell deformation: implications of the mechanical performance of *Arabidopsis hypocotyls* with pectin alterations. Mol. Plant 2, 990–999. 10.1093/mp/ssp065 19825674

[B2] Agu R. Devenny D. Tillett I. Palmer G. (2002). Malting performance of normal huskless and acid-dehusked barley samples. J. Inst. Brew. 108, 215–220. 10.1002/j.2050-0416.2002.tb00543.x

[B3] Baik B. K. Ullrich S. E. (2008). Barley for food: characteristics, improvement, and renewed interest. J. Cereal Sci. 48, 233–242. 10.1016/j.jcs.2008.02.002

[B4] Basu B. Tiwari D. Kundu D. Prasad R. (2009). Is Weibull distribution the most appropriate statistical strength distribution for brittle materials? Ceramics Int. 35, 237–246. 10.1016/j.ceramint.2007.10.003

[B5] Bidhendi A. J. Geitmann A. (2018). Tensile testing of primary plant cells and tissues, in Plant Biomechanics, eds Geitmann A. Gril J. (Cham: Springer), 321–347. 10.1007/978-3-319-79099-2_15

[B6] Brennan M. Hedley P. E. Topp C. F. E. Morris J. Ramsay L. Mitchell S.. (2019). Development and quality of barley husk adhesion correlates with changes in caryopsis cuticle biosynthesis and composition. Front. Plant Sci. 10:672. 10.3389/fpls.2019.00672 31178883 PMC6543523

[B7] Brennan M. Shepherd T. Mitchell S. Topp C. Hoad S. (2017a). Husk to caryopsis adhesion in barley is influenced by pre-and post-anthesis temperatures through changes in a cuticular cementing layer on the caryopsis. BMC Plant Biol. 17:169. 10.1186/s12870-017-1113-4 29058624 PMC5651604

[B8] Brennan M. Topp C. Hoad S. (2017b). Variation in grain skinning among spring barley varieties induced by a controlled environment misting screen. J. Agric. Sci. 155, 317–325. 10.1017/S0021859616000423

[B9] Brulé V. Rafsanjani A. Pasini D. Western T. L. (2016). Hierarchies of plant stiffness. Plant Sci. 250, 79–96. 10.1016/j.plantsci.2016.06.002 27457986

[B10] Burgert I. Keplinger T. (2013). Plant micro-and nanomechanics: experimental techniques for plant cell-wall analysis. J. Exp. Bot. 64, 4635–4649. 10.1093/jxb/ert255 24064925

[B11] Cavalier D. M. Lerouxel O. Neumetzler L. Yamauchi K. Reinecke A. Freshour G.. (2008). Disrupting two *Arabidopsis thaliana* xylosyltransferase genes results in plants deficient in xyloglucan, a major primary cell wall component. Plant Cell 20, 1519–1537. 10.1105/tpc.108.059873 18544630 PMC2483363

[B12] Chang K. H. (2014). Fatigue and Fracture Analysis. Oxford: Academic Press.

[B13] Chau K. Wei X. Wong R. Yu T. (2000). Fragmentation of brittle spheres under static and dynamic compressions: experiments and analyses. Mech. Mater. 32, 543–554. 10.1016/S0167-6636(00)00026-0

[B14] Collins H. Logue S. Jefferies S. Stuart I. Barr A. (1991). A study of the physical, biochemical and genetic factors influencing malt extract, in Proceedings of the 10th Australian Barley Technical Symposium (Canberra, ACT).

[B15] Duan R. Xiong H. Wang A. Chen G. (2015). Molecular mechanisms underlying hull-caryopsis adhesion/separation revealed by comparative transcriptomic analysis of covered/naked barley (*Hordeum vulgare* L.). *Int. J. Mol. Sci* 16, 14181–14193. 10.3390/ijms160614181 PMC4490547 26110389

[B16] Evers T. Millar S. (2002). Cereal grain structure and development: some implications for quality. J. Cereal Sci. 36, 261–284. 10.1006/jcrs.2002.0435

[B17] Faisal T. Rey A. Pasini D. (2013). A multiscale mechanical model for plant tissue stiffness. Polymers 5, 730–750. 10.3390/polym5020730

[B18] Fisher H. (1970). Growing barley for grain in Western Australia: varieties and production methods. J. Dept. Agric. 11, 93–97.

[B19] Fox G. (2008). Biochemical and molecular evaluation of quality for malt and feed barley (Ph.D. thesis), Southern Cross University, Lismore, NSW, Australia.

[B20] Fox G. P. Kelly A. M. Cakir M. Bloustein G. Poulsen D. M. Inkerman P. A. (2006). Genetic impacts of the hull on barley grain quality. J. Inst. Brew. 112, 101–107. 10.1002/j.2050-0416.2006.tb00238.x

[B21] Gaines R. Bechtel D. Pomeranz Y. (1985). A microscopic study on the development of a layer in barley that causes hull-caryopsis adherence. Cereal Chem. 62, 35–40.

[B22] Geitmann A. Gril J. (2018). Plant Biomechanics. Cham: Springer.

[B23] Ghanbarian B. Daigle H. (2015). Fractal dimension of soil fragment mass-size distribution: a critical analysis. Geoderma 245, 98–103. 10.1016/j.geoderma.2015.02.001

[B24] Gibson L. J. (2005). Biomechanics of cellular solids. J. Biomech. 38, 377–399. 10.1016/j.jbiomech.2004.09.027 15652536

[B25] Gibson L. J. (2012). The hierarchical structure and mechanics of plant materials. J. R. Soc. 9, 2749–2766. 10.1098/rsif.2012.0341 22874093 PMC3479918

[B26] Gorham D. Salman A. (2005). The failure of spherical particles under impact. Wear 258, 580–587. 10.1016/j.wear.2004.09.012

[B27] Grant K. R. (2019). Identifying Genotypic Risk Factors for Grain Skinning in Malting Barley. Ph.D. thesis, University of Edinburgh, UK.

[B28] Grant K. R. Brennan M. Hoad S. P. (in preparation). *Increasing Cuticle Permeability Drives Adhesion in the Barley Grain*.

[B29] Gubatz S. Dercksen V. J. Brüß C. Weschke W. Wobus U. (2007). Analysis of barley (*Hordeum vulgare*) grain development using three-dimensional digital models. Plant J. 52, 779–790. 10.1111/j.1365-313X.2007.03260.x 17825055

[B30] Gupta M. Abu-Ghannam N. Gallaghar E. (2010). Barley for brewing: characteristic changes during malting, brewing and applications of its by-products. Compreh. Rev. Food Sci. Food Saf. 9, 318–328. 10.1111/j.1541-4337.2010.00112.x 33467816

[B31] Harlan H. V. Martini M. L. (1936). Problems and results in barley breeding. Yearb. Agric. 1936, 303–346.

[B32] Hoad S. P. Brennan M. Wilson G. W. Cochrane P. M. (2016). Hull to caryopsis adhesion and grain skinning in malting barley: identification of key growth stages in the adhesion process. J. Cereal Sci. 68, 8–15. 10.1016/j.jcs.2015.10.007

[B33] Hoad S. P. Ellis R. P. Cochrane P. E. Thomas W. T. B. (2003). Causes and Control of Endosperm Exposure in Barley. Final Report 298. Technical report, Home Grown Cereal Authority (HGCA).

[B34] Huang S. Cerny R. E. Bhat D. S. Brown S. M. (2001). Cloning of an *Arabidopsis patatin*-like gene, *STURDY*, by activation T-DNA tagging. Plant Physiol. 125, 573–584. 10.1104/pp.125.2.573 11161015 PMC64859

[B35] Huang W. Zhang Y. Wang W. Zhang C. Binienda W. (2018). Numerical and experimental study on deformation and failure of trees under high-velocity impact loads. Earth Space 2018:637 10.1061/9780784481899.061

[B36] Jensen O. E. Fozard J. A. (2015). Multiscale models in the biomechanics of plant growth. Physiology 30, 159–166. 10.1152/physiol.00030.2014 25729061 PMC4346705

[B37] Kakeda K. Ishihara N. Izumi Y. Sato K. Taketa S. (2011). Expression and functional analysis of the barley *Nud* gene using transgenic rice. Breed. Sci. 61, 35–42. 10.1270/jsbbs.61.35

[B38] Kellogg E. A. (2001). Evolutionary history of the grasses. Plant Physiol. 125, 1198–1205. 10.1104/pp.125.3.1198 11244101 PMC1539375

[B39] Kinloch A. J. (2012). Adhesion and Adhesives: Science and Technology. Springer.

[B40] Kohl S. Hollmann J. Erban A. Kopka J. Riewe D. Weschke W.. (2015). Metabolic and transcriptional transitions in barley glumes reveal a role as transitory resource buffers during endosperm filling. J. Exp. Bot. 66, 1397–1411. 10.1093/jxb/eru492 25617470 PMC4339599

[B41] Köhler L. Spatz H.-C. (2002). Micromechanics of plant tissues beyond the linear-elastic range. Planta 215, 33–40. 10.1007/s00425-001-0718-9 12012239

[B42] Krifa M. (2009). A mixed Weibull model for size reduction of particulate and fibrous materials. Powder Technol. 194, 233–238. 10.1016/j.powtec.2009.04.011

[B43] Kruschke J. (2014). Doing Bayesian Data Analysis: A Tutorial With R, JAGS, and Stan. Cambridge, MA: Academic Press.

[B44] Kruschke J. K. Liddell T. M. (2018). The Bayesian new statistics: hypothesis testing, estimation, meta-analysis, and power analysis from a Bayesian perspective. Psychon. Bull. Rev. 25, 178–206. 10.3758/s13423-016-1221-4 28176294

[B45] Kumlehn J. Stein N. (2014). Biotechnological Approaches to Barley Improvement, Vol. 69, Berlin: Springer.

[B46] Kuo-Huang L.-L. Huang Y.-S. Chen S.-S. Huang Y.-R. (2004). Growth stresses and related anatomical characteristics in coconut palm trees. IAWA J. 25, 297–310. 10.1163/22941932-90000367

[B47] Laidig F. Piepho H.-P. Rentel D. Drobek T. Meyer U. (2017). Breeding progress, genotypic and environmental variation and correlation of quality traits in malting barley in German official variety trials between 1983 and 2015. Theor. Appl. Genet. 130, 2411–2429. 10.1007/s00122-017-2967-4 28821914 PMC5641284

[B48] Lemloh M.-L. Pohl A. Weber E. Zeiger M. Bauer P. Weiss I. M. (2014). Structure-property relationships in mechanically stimulated *Sorghum* bicolor stalks. Bioinsp. Mater. 1, 1–11. 10.2478/bima-2014-0001

[B49] López-Perea P. Schwarz P. Figueroa J. Hernández-Estrada Z. (2012). Effect of β-glucans on viscoelastic properties of barley kernels and their relationship to structure and soluble dietary fibre. J. Cereal Sci. 56, 595–602. 10.1016/j.jcs.2012.07.017

[B50] Lu C. Danzer R. Fischer F. D. (2002). Fracture statistics of brittle materials: Weibull or normal distribution. Phys. Rev. 65:067102 10.1103/PhysRevE.65.067102 12188868

[B51] Luo Q. Zhou K. Zhao X. Zeng Q. Xia H. Zhai W.. (2005). Identification and fine mapping of a mutant gene for palealess spikelet in rice. Planta 221, 222–230. 10.1007/s00425-004-1438-8 15605239

[B52] Masselter T. Speck T. (2011). Biomimetic fiber-reinforced compound materials, in Advances in Biomimetics, ed George A. (Rijeka: InTech), 185–210. 10.5772/14899

[B53] Meredith W. (1959). Note on the malting quality of peeled barley. J. Inst. Brew. 65, 31–33. 10.1002/j.2050-0416.1959.tb01423.x

[B54] Niklas K. J. (1992). Plant Biomechanics: An Engineering Approach to Plant Form and Function. Chicago, IL: University of Chicago Press.

[B55] Niklas K. J. (1999). A mechanical perspective on foliage leaf form and function. New Phytol. 143, 19–31. 10.1046/j.1469-8137.1999.00441.x

[B56] Niklas K. J. Paolillo D. J. Jr. (1997). The role of the epidermis as a stiffening agent in *Tulipa* (liliaceae) stems. Am. J. Bot. 84, 735–744. 10.2307/2445809 21708626

[B57] Okoro P. Brennan M. Bryce J. Smith P. Kelly H. Hoad S. (2017). Effects of grain skinning on the malting performance of barley, in Worldwide Distilled Spirits Conference (Glasgow).

[B58] Olkku J. Salmenkallio-Marttila M. Sweins H. Home S. (2005). Connection between structure and quality of barley husk. J. Am. Soc. Brew. Chem. 63, 17–22. 10.1094/ASBCJ-63-0017

[B59] Olmedo I. Bourrier F. Bertrand D. Berger F. Limam A. (2018). Dynamic analysis of wooden rockfall protection structures subjected to impact loading using a discrete element model. Eur. J. Environ. Civil Eng. 24, 1430–1449. 10.1080/19648189.2018.1472042

[B60] Paluszny A. Tang X. Nejati M. Zimmerman R. W. (2016). A direct fragmentation method with Weibull function distribution of sizes based on finite-and discrete element simulations. Int. J. Solids Struct. 80, 38–51. 10.1016/j.ijsolstr.2015.10.019

[B61] Paul-Victor C. Rowe N. (2010). Effect of mechanical perturbation on the biomechanics, primary growth and secondary tissue development of inflorescence stems of *Arabidopsis thaliana. Ann. Bot* 107, 209–218. 10.1093/aob/mcq227 PMC3025729 21118840

[B62] Perfect E. (1997). Fractal models for the fragmentation of rocks and soils: a review. Eng. Geol. 48, 185–198. 10.1016/S0013-7952(97)00040-9

[B63] Plummer M. (2016). rjags: Bayesian Graphical Models Using MCMC. R package version 4–6. CRAN. Available online at: https://CRAN.R-project.org/package=rjags

[B64] Pozzi C. Faccioli P. Terzi V. Stanca A. M. Cerioli S. Castiglioni P.. (2000). Genetics of mutations affecting the development of a barley floral bract. Genetics 154, 1335–1346. Available online at: https://www.genetics.org/content/154/3/1335.short 10757774 10.1093/genetics/154.3.1335 PMC1460976

[B65] Psota V. Hartmann J. Sejkorová Š. Loučková T. Vejražka K. (2009). 50 years of progress in quality of malting barley grown in the Czech Republic. J. Inst. Brew. 115, 279–291. 10.1002/j.2050-0416.2009.tb00382.x

[B66] R Core Team (2018). R: A Language and Environment for Statistical Computing. Vienna: R Foundation for Statistical Computing.

[B67] Rajasekaran P. Thomas W. Wilson A. Lawrence P. Young G. Ellis R. (2004). Genetic control over grain damage in a spring barley mapping population. Plant Breed. 123, 17–23. 10.1046/j.0179-9541.2003.00913.x

[B68] Reinbergs E. Huntley D. (1957). Some factors affecting hull adherence in barley. Can. J. Plant Sci. 37, 262–273. 10.4141/cjps57-032

[B69] Robertson D. J. Smith S. L. Cook D. D. (2015). On measuring the bending strength of septate grass stems. Am. J. Bot. 102, 5–11. 10.3732/ajb.1400183 25587143

[B70] Roumeliotis S. Collins H. Logue S. Willsmore K. Jefferies S. Barr A. (1999). Implications of thin husk in barley, in Proceeding of the Ninth Australian Barley Technical Symposium (Melbourne, VIC).

[B71] Roumeliotis S. Eglinton J. (2007). Response to selection for increased malt extract, in Proceedings of the 13th Australian Barley Technical Symposium (Perth, WA), 83–92.

[B72] Rüggeberg M. Burgert I. Speck T. (2009). Structural and mechanical design of tissue interfaces in the giant reed Arundo donax. J. R. Soc. Interface 7, 499–506. 10.1098/rsif.2009.0273 19726440 PMC2842797

[B73] Rüggeberg M. Speck T. Paris O. Lapierre C. Pollet B. Koch G. (2008). Stiffness gradients in vascular bundles of the palm *Washingtonia robusta. Proc. R. Soc. B Biol. Sci* 275, 2221–2229. 10.1098/rspb.2008.0531 PMC2603245 18595839

[B74] Salman A. Reynolds G. Fu J. Cheong Y. Biggs C. Adams M. (2004). Descriptive classification of the impact failure modes of spherical particles. Powder Technol. 143, 19–30. 10.1016/j.powtec.2004.04.005

[B75] Sanchidrián J. A. Ouchterlony F. Moser P. Segarra P. López L. M. (2012). Performance of some distributions to describe rock fragmentation data. Int. J. Rock Mech. Mining Sci. 53, 18–31. 10.1016/j.ijrmms.2012.04.001

[B76] Sanchidrián J. A. Ouchterlony F. Segarra P. Moser P. (2014). Size distribution functions for rock fragments. Int. J. Rock Mech. Mining Sci. 71, 381–394. 10.1016/j.ijrmms.2014.08.007

[B77] Santurbano R. Fairhurst C. (1991). Fracture mechanics in the context of rock crushing: preliminary experimental results concerning the impact of limestone spheres, in TThe 32nd US Symposium on Rock Mechanics (USRMS) (Norman, OK: American Rock Mechanics Association). 10.1016/0148-9062(92)90804-9

[B78] Schindelin J. Arganda-Carreras I. Frise E. Kaynig V. Longair M. Pietzsch T.. (2012). Fiji: an open-source platform for biological-image analysis. Nat. Methods 9:676. 10.1038/nmeth.2019 22743772 PMC3855844

[B79] Seidel R. Thielen M. Schmitt C. Bührig-Polaczek A. Fleck C. Speck T. (2010). Fruit walls and nut shells as an inspiration for the design of bio-inspired impact resistant hierarchically structured materials. Des. Nat. 8, 421–430. 10.2495/DN100371

[B80] Shah D. U. Reynolds T. P. Ramage M. H. (2017). The strength of plants: theory and experimental methods to measure the mechanical properties of stems. J. Exp. Bot. 68, 4497–4516. 10.1093/jxb/erx245 28981787

[B81] Shockey D. A. Curran D. R. Seaman L. Rosenberg J. T. Petersen C. F. (1974). Fragmentation of rock under dynamic loads. Int. J. Rock Mech. Mining Sci. Geomech. Abstr. 11, 303–317. 10.1016/0148-9062(74)91760-4

[B82] Sieber P. Schorderet M. Ryser U. Buchala A. Kolattukudy P. Métraux J.-P.. (2000). Transgenic *Arabidopsis* plants expressing a fungal cutinase show alterations in the structure and properties of the cuticle and postgenital organ fusions. Plant Cell 12, 721–737. 10.1105/tpc.12.5.721 10810146 PMC139923

[B83] Smirnova A. Leide J. Riederer M. (2013). Deficiency in a very-long-chain fatty acid β-ketoacyl-coenzyme a synthase of tomato impairs microgametogenesis and causes floral organ fusion. Plant Physiol. 161, 196–209. 10.1104/pp.112.206656 23144186 PMC3532251

[B84] Spatz H.-C. Beismann H. Brüchert F. Emanns A. Speck T. (1997). Biomechanics of the giant reed *Arundo donax. Philos. Trans. R. Soc. Lond. B Biol. Sci* 352, 1–10. 10.1098/rstb.1997.0001

[B85] Speck T. Burgert I. (2011). Plant stems: functional design and mechanics. Annu. Rev. Mater. Res. 41, 169–193. 10.1146/annurev-matsci-062910-100425

[B86] Stubbs C. J. Baban N. S. Robertson D. J. Alzube L. Cook D. D. (2018). Bending stress in plant stems: models and assumptions, in Plant Biomechanics, eds Geitmann A. Gril J. (Cham: Springer), 49–77. 10.1007/978-3-319-79099-2_3

[B87] Taketa S. Amano S. Tsujino Y. Sato T. Saisho D. Kakeda K.. (2008). Barley grain with adhering hulls is controlled by an ERF family transcription factor gene regulating a lipid biosynthesis pathway. Proc. Natl. Acad. Sci. U.S.A. 105, 4062–4067. 10.1073/pnas.0711034105 18316719 PMC2268812

[B88] Tavakoli H. Mohtasebi S. Rajabipour A. Tavakoli M. (2009). Effects of moisture content, loading rate, and grain orientation on fracture resistance of barley grain. Res. Agric. Eng. 55, 85–93. 10.17221/6/2009-RAE

[B89] Tottman D. (1987). The decimal code for the growth stages of cereals, with illustrations. Ann. Appl. Biol. 110, 441–454. 10.1111/j.1744-7348.1987.tb03275.x

[B90] Turcotte D. (1986). Fractals and fragmentation. J. Geophys. Res. Solid Earth 91, 1921–1926. 10.1029/JB091iB02p01921

[B91] Vincent J. F. (1982). The mechanical design of grass. J. Mater. Sci. 17, 856–860. 10.1007/BF00540384

[B92] Vogel J. (2008). Unique aspects of the grass cell wall. Curr. Opin. Plant Biol. 11, 301–307. 10.1016/j.pbi.2008.03.002 18434239

[B93] Vogler H. Felekis D. Nelson B. Grossniklaus U. (2015). Measuring the mechanical properties of plant cell walls. Plants 4, 167–182. 10.3390/plants4020167 27135321 PMC4844320

[B94] Wang X. Ren H. Zhang B. Fei B. Burgert I. (2011). Cell wall structure and formation of maturing fibres of moso bamboo (*Phyllostachys pubescens*) increase buckling resistance. J. R. Soc. Interface 9, 988–996. 10.1098/rsif.2011.0462 21920959 PMC3306637

[B95] Weibull W. (1951). A statistical distribution function of wide applicability. J. Appl. Mech. 18, 293–297.

[B96] Wright C. T. Pryfogle P. A. Stevens N. A. Steffler E. D. Hess J. R. Ulrich T. H. (2005). Biomechanics of wheat/barley straw and corn stover. Appl. Biochem. Biotechnol. 121, 5–19. 10.1007/978-1-59259-991-2_2 15917584

[B97] Xu Y. Song D. Chu F. (2016). Approach to the weibull modulus based on fractal fragmentation of particles. Powder Technol. 292, 99–107. 10.1016/j.powtec.2016.01.021

[B98] Yeats T. H. Rose J. K. (2013). The formation and function of plant cuticles. Plant Physiol. 163, 5–20. 10.1104/pp.113.222737 23893170 PMC3762664

[B99] Yephremov A. Wisman E. Huijser P. Huijser C. Wellesen K. Saedler H. (1999). Characterization of the *FIDDLEHEAD* gene of *Arabidopsis* reveals a link between adhesion response and cell differentiation in the epidermis. Plant Cell 11, 2187–2201. 10.1105/tpc.11.11.2187 10559443 PMC144117

[B100] Yuan Z. Gao S. Xue D.-W. Luo D. Li L.-T. Ding S.-Y.. (2009). RETARDED PALEA1 controls palea development and floral zygomorphy in rice. Plant Physiol. 149, 235–244. 10.1104/pp.108.128231 18952859 PMC2613737

[B101] Zhong R. Taylor J. J. Ye Z.-H. (1997). Disruption of interfascicular fiber differentiation in an *Arabidopsis* mutant. Plant Cell 9, 2159–2170. 10.1105/tpc.9.12.2159 9437861 PMC157065

[B102] Zhong R. Ye Z.-H. (2004). Amphivasal Vascular Bundle 1, a gain-of-function mutation of the IFL1/REV gene, is associated with alterations in the polarity of leaves, stems and carpels. Plant Cell Physiol. 45, 369–385. 10.1093/pcp/pch051 15111711

[B103] Zhuang Z. Liu Z. (2014a). Dynamic Crack Propagation. Oxford: Academic Press.

[B104] Zhuang Z. Liu Z. (2014b). Fundamental Linear Elastic Fracture Mechanics. Oxford: Academic Press.

